# Correction: Morphological and Molecular Revision of the Genus *Ozirhincus* (Diptera: Cecidomyiidae)—Long-Snouted Seed-Feeding Gall Midges on Asteraceae

**DOI:** 10.1371/journal.pone.0135201

**Published:** 2015-08-05

**Authors:** The PLOS ONE Staff

## Notice of Republication

This article was republished on July 21, 2015, to replace the legend for Table 2, which was mistakenly published as body text, to include the photo credit in the caption of Fig 2, and to correct the formatting of the species names in the Descriptions section of the Results. The publisher apologizes for the errors. Please download this article again to view the correct version. The originally published, uncorrected article and the republished, corrected article are provided here for reference.

## Supporting Information

S1 File Originally published, uncorrected article. (PDF)

S2 File Republished, corrected article. (PDF)

## References

[1] Dorchin N, Astrin JJ, Bodner L, Harris KM (2015) Morphological and Molecular Revision of the Genus *Ozirhincus* (Diptera: Cecidomyiidae)—Long-Snouted Seed-Feeding Gall Midges on Asteraceae. PLoS ONE 10(7): e0130981 doi: 10.1371/journal.pone.0130981 26134526 10.1371/journal.pone.0130981 PMC4489628

