# Supplementary material for: Correction: Morphological and Molecular Revision of the Genus Ozirhincus (Diptera: Cecidomyiidae)—Long-Snouted Seed-Feeding Gall Midges on Asteraceae
Source: PLoS One. 2015 Aug 5;10(8):e0135201. doi: 10.1371/journal.pone.0135201 (PMC4526538; doi:10.1371/journal.pone.0135201)
Supplement: S1 File — (PDF) [file pone.0135201.s001.pdf]

PLOS | ONE

RESEARCH ARTICLE

Morphological and Molecular Revision of the Genus *Ozirhincus* (Diptera: Cecidomyiidae)—Long-Snouted Seed-Feeding Gall Midges on Asteraceae

Netta Dorchin[1]*, Jonas J. Astrin[2], Levona Bodner[1], Keith M. Harris[3]

1 Department of Zoology, Tel Aviv University, Tel Aviv, Israel, **2** Alexander Koenig Zoological Research Museum, Bonn, Germany, **3** Independent researcher, Ripley, United Kingdom

* ndorchin@post.tau.ac.il

CrossMark
click for updates

OPEN ACCESS

Citation: Dorchin N, Astrin JJ, Bodner L, Harris KM (2015) Morphological and Molecular Revision of the Genus *Ozirhincus* (Diptera: Cecidomyiidae)—Long-Snouted Seed-Feeding Gall Midges on Asteraceae. PLoS ONE 10(7): e0130981. doi:10.1371/journal.pone.0130981

Editor: Sean Brady, Smithsonian National Museum of Natural History, UNITED STATES

Received: January 24, 2015

Accepted: May 26, 2015

Published: July 2, 2015

Copyright: © 2015 Dorchin et al. This is an open access article distributed under the terms of the Creative Commons Attribution License, which permits unrestricted use, distribution, and reproduction in any medium, provided the original author and source are credited.

Data Availability Statement: All relevant data are within the paper and its Supporting Information files. Sequences are available from the GenBank database as detailed in the manuscript.

Funding: ND received a grant from the Israeli Taxonomy Initiative (ITI) (http://taxonomy.tau.ac.il/en/). Museum Alexander Koenig provided funds to JJA for molecular work (www.zfmk.de). The funders had no role in study design, data collection and analysis, decision to publish, or preparation of the manuscript.

Abstract

The Palaearctic gall-midge genus *Ozirhincus* is unique among the Cecidomyiidae for its morphology and biology. Unlike most other phytophagous gall midges, species in this genus do not induce galls but develop inside achenes of Asteraceae plants. The heads of adults are characterized by an unusually elongate proboscis, the function of which is unclear. Despite a lot of attention from taxonomists in the 19th and early 20th century, a proper revision of the genus has been hindered by complex host associations, the loss of most relevant type material, and the lack of a thorough comparative study of all life stages. The present revision integrated morphological, molecular, and life-history data to clearly define species boundaries within *Ozirhincus*, and delimit host-plant ranges for each of them. A phylogenetic analysis based on the mitochondrial COI and 16S genes confirmed the validity of four distinct species but did not resolve the relationships among them. All species are oligophages, and some may occur together on the same host plant. Species with wider host-plant ranges have wider European and circum-Mediterranean distribution ranges, whereas species with narrower host ranges are limited to Europe and the Russian Far East. As part of the present work, *O. hungaricus* is reinstated from synonymy, *O. tanaceti* is synonymized under *O. longicollis*, neotypes are designated for *O. longicollis* and *O. millefolii*, and a lectotype is designated for *O. anthemidis*.

Introduction

Gall midges (Cecidomyiidae) constitute one of the largest families of Diptera, with more than 6200 described species and many that are undescribed or unknown [1]. Most of these species are plant feeders in the larval stage, and induce galls, the complexity and morphological diversity of which is rivalled only by gall wasps (Hymenoptera: Cynipidae) [2], [3]. Despite the ubiquity of gall midges and their fascinating biology, the family is considered to be taxonomically difficult because of the relative scarcity of useful characters, the small size of the midges, and

Competing Interests: The authors have declared that no competing interests exist.

the fact that their study requires special techniques. Knowledge about gall midge systematics is lacking, and many genera are in need of taxonomic revision or even placement to tribe [1]. Such revisions ideally combine morphological, molecular, and life-history data, as this approach produces the most informative and reliable taxonomy, and may offer insight into the evolution of the studied groups.

Ozirhincus Rondani is a small cecidomyiid genus that is unique for its morphology and biology. Adults in this genus differ from other cecidomyiids in having an unusually elongate proboscis and flattened head, and for developing in achenes of Asteraceae rather than inducing galls, as do most other members of the tribe Lasiopterini [4], [1]. Members of this genus are known only from host plants of the tribe Anthemideae and are restricted to the Old World, with the exception of *O. millefolii*, which was introduced into North America in colonial times [5], [4]. Based on its morphology and biology, the genus belongs to the subtribe Lasiopterina [6], and is apparently closely related to *Lasioptera*, one of the largest and most loosely defined genera of Cecidomyiidae [4]. The unique morphological characters exhibited by *Ozirhincus* species, and their specialized life history, strongly suggest that the genus constitutes a monophyletic group.

The genus has drawn the attention of many researchers since it was first described by Rondani in 1840 [7], as attested by unusual nomenclatorial vicissitudes (reviewed by Verrall, [8]), but despite this attention, its taxonomy remained confused until now. A major difficulty stemmed from the fact that *Ozirhincus* species appeared to have a wide range of host plants, and that some of these hosts support more than one species of gall midge at the same time and place [9–12]. An added complexity was introduced by Möhn's revision of *Ozirhincus* [11], [12], which was based solely on larvae that were dissected from dried herbarium material. In that revision, Möhn synonymized 5 of the previously known species and described 7 new ones, basing all taxonomic decisions on problematic morphological characters of the mostly uninformative larvae. Recognizing that larval characters show high intraspecific variability and low interspecific differences, and based on collecting efforts throughout Europe, Skuhravá [13] reversed most of Möhn's decisions, leaving *Ozirhincus* with 5 valid species, though without clear characters to distinguish between them. The fact that most historical types of species described in the 19th century are considered lost, led to further complications for clarifying the taxonomy of this genus.

The objective of the present study was to settle the taxonomy of *Ozirhincus* through extensive sampling from known and potential host plants and the combination of morphological and molecular data. Our specific goals were: 1) to identify reliable morphological characters for distinguishing between the species in the genus based on a thorough study of adults, pupae, and larvae, 2) to clarify the complex host associations in the genus, and 3) to produce a phylogeny for the genus based on genetic markers.

Materials and Methods

Collecting and rearing of insects

Gall midges in the genus *Ozirhincus* do not cause the formation of galls, therefore rearing them from their host plants requires collection of normal-looking inflorescences of potential host plants towards the end of flowering, and keeping them in rearing cages in the laboratory until adult emergence. In this study we screened 27 potential host plants (Table 1) in Germany, Israel, and the UK, based on host records from the literature and the fact that *Ozirhincus* is known only from plants of the tribe Anthemideae [1], [11, 12]. *Ozirhincus millefolii* was also collected in the USA, where it is widespread on its main host plant, *Achillea millefolium*, which had been introduced from Europe, probably in colonial times [4]. In the following species

Table 1. Potential Asteraceae host plants screened in this study for the presence of *Ozirhincus* gall midges.

Plant	Asteraceae tribe	Confirmed as a host?
Aaronsonia factorovskyi	Anthemideae	-
Achillea fragrantissima	Anthemideae	-
Achillea millefolium	Anthemideae	+
Achillea ptarmica	Anthemideae	+
Achillea santolina	Anthemideae	-
Anthemis bornmuelleri	Anthemideae	+
Anthemis cotula	Anthemideae	+
Anthemis palestina	Anthemideae	-
Anthemis pseudocotula	Anthemideae	+
Anthemis rascheyana	Anthemideae	+
Anthemis retusa	Anthemideae	+
Anthemis tinctoria	Anthemideae	+
Artemisia arborescence	Anthemideae	-
Artemisia judaica	Anthemideae	-
Artemisia monosperma	Anthemideae	-
Artemisia sieberi	Anthemideae	-
Bellis perrenis	Astereae	-
Chrysanthemum coronarium	Anthemideae	+
Chrysanthemum segetum	Anthemideae	+
Erigeron sp.	Astereae	-
Leucanthemum vulgare	Anthemideae	+
Matricaria aurea	Anthemideae	-
Matricaria recutita	Anthemideae	-
Tanacetum parthenium	Anthemideae	+
Tanacetum santolinoides	Anthemideae	-
Tanacetum vulgare	Anthemideae	+
Tripleurospermum inodorum	Anthemideae	+

doi:10.1371/journal.pone.0130981.t001

descriptions, only those names of plants that were confirmed as hosts in the present study are given. Many other plant species have been mentioned in the literature but were not confirmed here. Some of them were sampled in the present work but did not yield gall midges; for those that were not sampled in the present study, further sampling and rearing will be needed to confirm their host status because host records were made based on larvae alone [11], [12], and it is impossible to say to which *Ozirhincus* species those larvae belonged.

Inflorescences were collected in the field and transferred to the laboratory in large plastic bags. They were then either kept in sealed bags, or placed as bouquets in water in ventilated rearing cages until adult gall midges emerged from them or until they wilted without producing gall midges. Plant species that did not yield gall midges were usually re-sampled several times during their flowering season, sometimes in two or three consecutive years and at several localities, to confirm that they are not hosts. Some inflorescences were dissected under a stereomicroscope to obtain larvae and pupae for morphological study.

To establish the monophyly of *Ozirhincus*, we chose outgroups representing four genera from the two subtribes of Lasiopterini, the tribe to which *Ozirhincus* belongs. The subtribe Lasiopterina is represented by *Lasioptera*, a large, cosmopolitan genus that is closely related to *Ozirhincus* based on morphological characters, whereas the subtribe Baldratiina, which is

restricted to host plants of the Chenopodiaceae is represented by the genera *Baldratia*, *Careopalpis*, and *Stefaniola*. All methods described for *Ozirhincus* apply also to the outgroups.

Molecular methods

Genomic DNA was extracted from whole adult or immature midges using mostly individual silica-membrane columns from the Blood and Tissue kit by Qiagen (Hilden, Germany). DNA extracts are available from the ZFMK Biobank, Bonn (DNA voucher IDs are given in Appendix A). For PCR amplifications, we used the Qiagen Multiplex PCR kit, following the manufacturer's specifications and based on 2–2.5µl undiluted DNA template in 20µl total reaction volumes. The GeneAmp PCR System 2700 (Applied Biosystems, Foster City, CA, USA) was used to amplify two mitochondrial genes. A 658 bp fragment from the 5' part of the mitochondrial cytochrome oxidase subunit I (COI) gene (the so called 'DNA barcoding gene') was amplified using the primers LCO1490 (F) and HCO2198 (R) [14]. Failed reactions were repeated with the primer combination LCO1490 (F) and C1-N-2191 (R, aka 'Nancy') [15]. A 532–533 bp fragment from the 3' end of the 16S ribosomal RNA gene was amplified using the primers 16S-ar-JJ and 16S-1472-JJ [16]. For both genes, the touchdown PCR protocol started at an annealing temperature of 55°C, decreasing to 40°C for the remaining 25 cycles. Following enzymatic clean-up (Exo/SAP), double-stranded sequencing was conducted on an automated ABI 3730XL sequencer (Applied Biosystems) at the Macrogen facility, Amsterdam, NL. Sequences were assembled, inspected and aligned using Geneious vers. R7 (Biomatters, Auckland, New Zealand). The COI + 16S datasets were concatenated in BioEdit [17], resulting in a combined alignment of 1191 bp. Sequences are deposited in GenBank (http://www.ncbi.nlm.nih.gov/) and accession numbers are provided in Appendix A.

Phylogenetic analysis

MODELTEST ver. 3.7 [18] consistently identified the GTR+G model of nucleotide substitution [19] as the best-fit model for the COI data. For 16S, heterogeneous models were suggested by different algorithms (hierarchical likelihood ratio test, Akaike information criterion, Bayesian information criterion) and conditions (Bayesian information criterion), but most converged on assuming a number of substitution types of 6, so that the same model was applied as for COI. Bayesian analyses were conducted using MrBayes vers. 3.2.0 [20]. Specific parameters for the GTR+G model were equated by MrBayes. Parameters were unlinked between the 3rd versus 1st plus 2nd codon positions and for 16S. We ran two independent replicates (4 chains each) for 70 million generations per analysis. Every 1000th tree was sampled. Negative log-likelihood score stabilization was determined in a separate visualization and the trees for the first 19.000 generations were discarded as burn-in accordingly. We thus retained 139,962 trees, which were used for building a 50%-majority rule consensus tree with posterior probabilities.

We reconstructed the evolution of proboscis length as an unordered multistate character onto our phylogenetic tree using the maximum parsimony approach, as implemented in MESQUITE vers. 3.03 [21].

Taxonomy

Larvae, exuviae, and adults of the gall midges were preserved in 70% ethanol for morphological study, and were later mounted on permanent microscope slides in euparal according to the method outlined by Gagné [22]. Specimens are mounted on slides individually unless otherwise noted. Relevant historical material from the Rübsaamen, and Möhn collections was examined, and its condition evaluated. Material in these collections had originally been mounted on temporary microscope slides in glycerin, or stored in 70% ethanol, and many of the specimens

are either lost or have deteriorated to the point that they cannot be used for taxonomic study. Some of that material that was found to be in reasonable condition was remounted on permanent microscope slides in euparal for the purpose of the present study and to ensure its integrity and long-term preservation.

Illustrations of morphological structures were made with the aid of a drawing tube or a Leica DFC295 camera mounted on a Leica DM1000 LED compound microscope. Pupae were studied under a scanning electron microscope. Some adults were also pinned to preserve the color pattern created by the thick covering of scales particularly on the abdomen. Proboscis length was measured from the suture at the base of the labrum to the tip of the labella, and expressed in relation to the length of the antennal scape. Ovipositor length was measured from the base of the eighth abdominal segment to the apex of the cercus, and expressed in relation to the length of segment 8. Some adults and immature stages were preserved in 96% ethanol for molecular study. We compared newly collected material to types and other relevant material deposited in the Staatliches Museum für Naturkunde, Stuttgart, Germany (SMNS), Museum für Naturkunde, Berlin, Germany (ZMHB), the Naturhistorisches Museum, Vienna, Austria (NHMW), the Natural History Museum in London, UK (BMNH), and the private collections of Marcela Skuhravá (Prague) and Eddy Dijktra (Delft). Terminology for adult morphology follows McAlpine et al. [23], and terminology for immature morphology follows Gagné [4]. The specimens examined in this work are deposited in the National Collection of Insects, Zoological Museum, Tel Aviv University (TAUI) unless otherwise indicated.

Results

Phylogenetic analysis

The complete molecular dataset of COI and 16S consisted of 1191 bp. Unaligned fragment lengths for 16S were 532–533 bp, i.e. included only a single-base indel. The COI fragment consisted of 658 positions and contained no internal gaps. The phylogenetic analysis yielded four strongly supported and genetically quite distant clades, clearly representing four valid species in the genus *Ozirhincus* (Fig 1), which can be distinguished from each other based on unique combinations of larval, pupal, and adult morphological characters. However, the analysis could not resolve the phylogenetic relationships among the four species. The ancestral states analysis did not yield conclusive results regarding the history of proboscis length in the genus (see character states in Fig 1). It is likely that the short proboscis in *O. anthemidis* represents the ancestral state, similar to the situation in *Lasioptera*, and that the typical elongate proboscis evolved only once in the history of the genus, but other scenarios cannot be ruled out.

Mapping the identity of host-plant genera on the phylogenetic tree (colors in Fig 1) suggests that *O. millefolii* is restricted to the genus *Achillea*, whereas each of the remaining species is capable of developing in host plants of various genera. It also indicates that certain host plants support more than one *Ozirhincus* species at the same time and place: *Tripleurospermum inodorum* is used by *O. longicollis* and *O. hungaricus*, while some *Anthemis* spp. are hosts to both *O. longicollis* and *O. anthemidis*. In all of these scenarios it is possible to differentiate among the gall-midge species based at least on their adult and pupal morphology.

Taxonomy

Ozirhincus Rondani 1840

Type species: *Ozirhincus longicollis* Rondani 1840: 16; by monotypy

Ozirhincus is a small genus in the tribe Lasiopterini, apparently closely related to *Lasioptera*. Like other Lasiopterini, it is characterized by a thick covering of scales (Fig 2A–2C), an irregular number of antennal flagellomeres that are gynecoid in the male, a very short R_{4+5} that

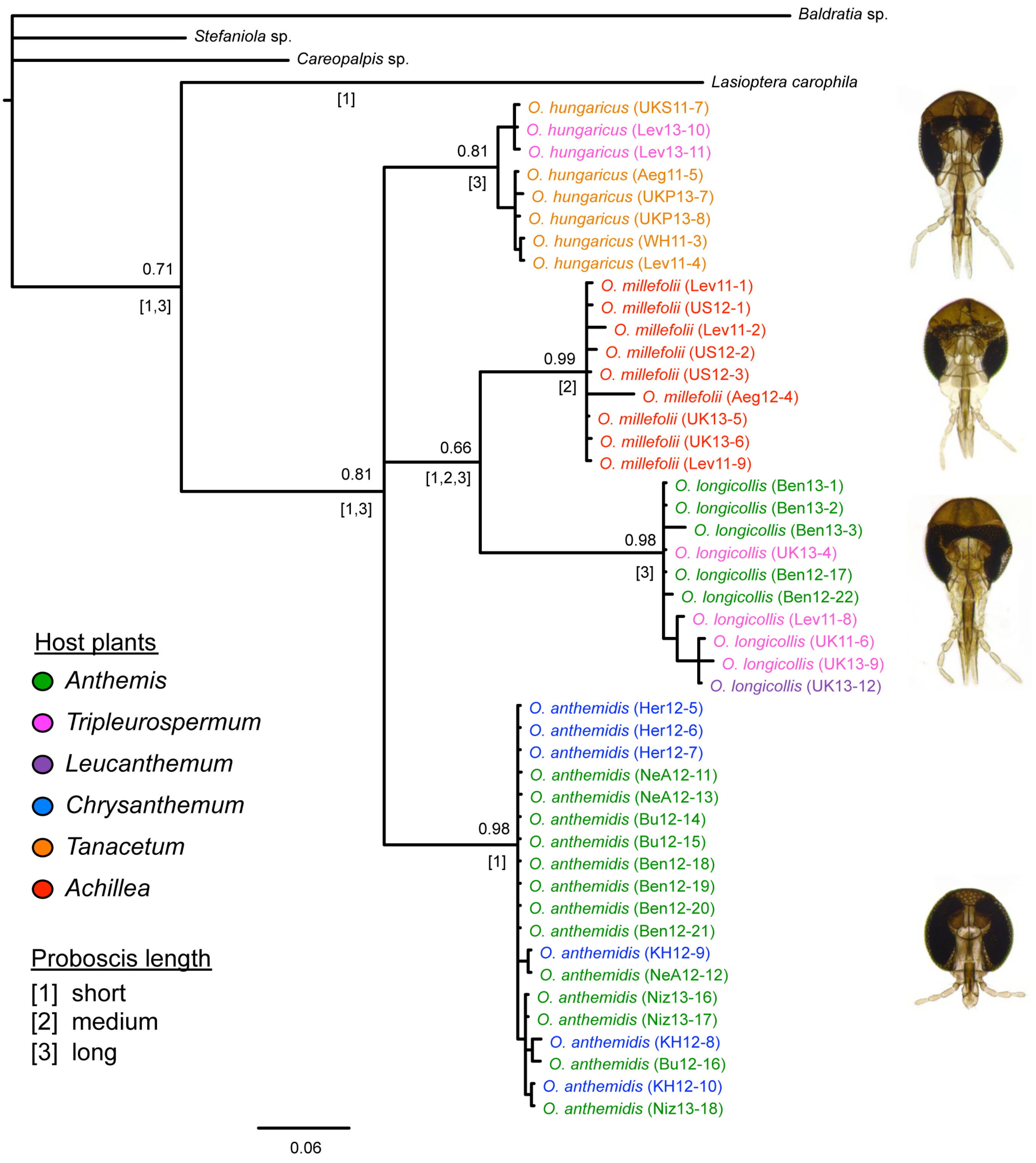

Fig 1. Phylogenetic tree of *Ozirhincus* Rondani based on Bayesian analysis of partial sequence of the cytochrome oxidase subunit I (COI) and ribosomal RNA16S mitochondrial genes. Support values are shown next to nodes, above branches. Character states representing proboscis length as suggested by the ancestral states analysis are shown below branches (in square brackets). Letters and numbers following species name refer to collecting localities and dates (details in Appendix A). Colors correspond to host-plant genera.

doi:10.1371/journal.pone.0130981.g001

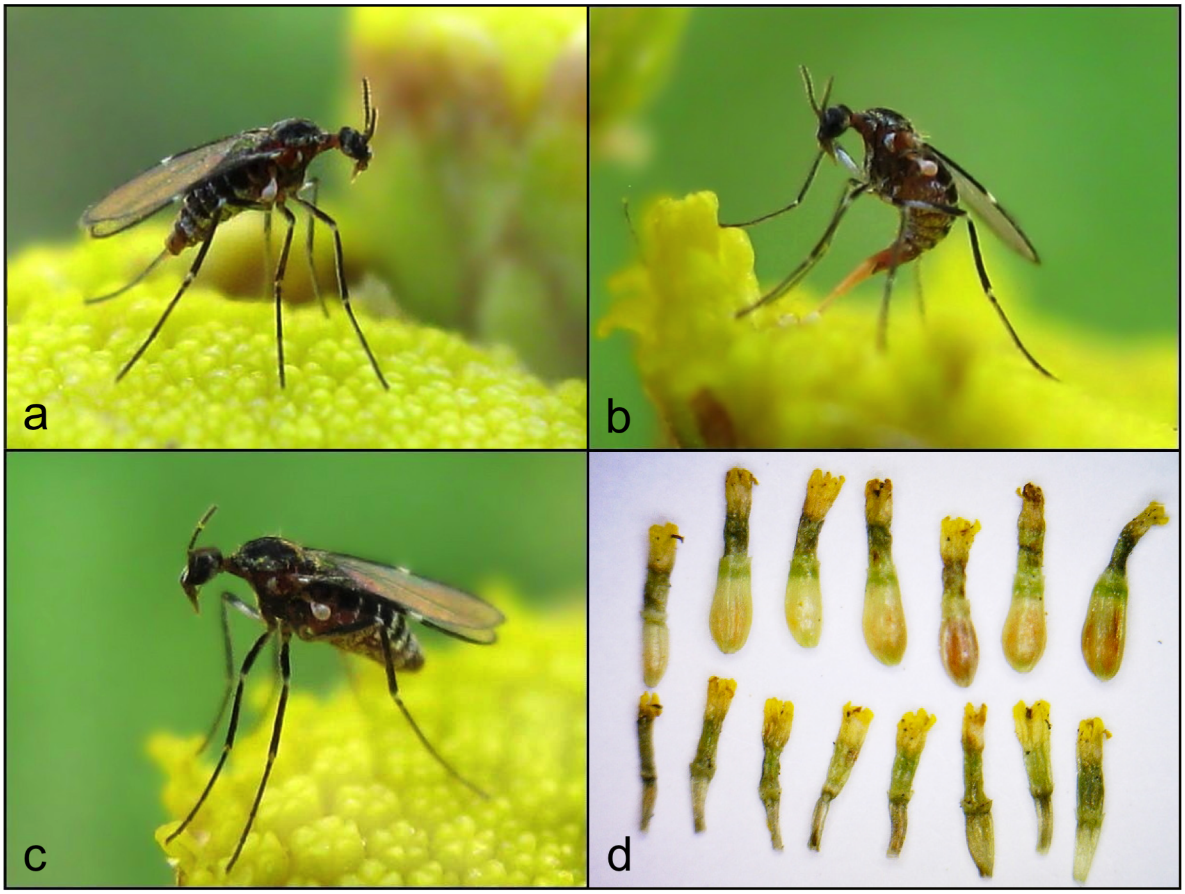

Fig 2. *Ozirhincus hungaricus*. a-c. Female on *Tanacetum vulgare* inflorescence; d. *Tanacetum vulgare* flowers containing *O*. *hungaricus* larvae (upper row), and normal flowers (lower row).

doi:10.1371/journal.pone.0130981.g002

reaches C proximal to mid-length of wing, longitudinally divided mediobasal lobes in the male that sheath the aedeagus almost to its apex, a protractible ovipositor with a lateral group of hooked setae on segment 8 of the female abdomen, and variously modified setae on the cercal segment. In the closely related *Lasioptera*, it was argued that these modified setae in the female function in collecting and carrying conidia [24], [25], but their function in *Ozirhincus* is unclear because species in this genus do not appear to be associated with fungal symbionts. Adults in this genus have unusually elongate mouthparts that form a short to very long proboscis, composed mostly of the strongly setose labrum and labella, and a considerable elongation of the frontoclypeal membrane (Fig 3A–3E). The head in most species is flattened, with the occiput encroaching the eye area. Palps are four-segmented. Tarsal claws are toothed on all legs. The body is covered by black and white scales that form dorsal transverse stripes on the abdomen. Larvae have a bifid spatula in the third instar, with a reduced number of 3–4 lateral asetose papillae on each side, and usually 2–3 setose papillae on each side of the terminal segment. Pupae have pointed antennal bases ('antennal horns') that terminate in one or two tips. All species develop in achenes of Asteraceae belonging to the tribe Anthemideae (Fig 2D). Pupation takes place inside the achene and the species complete at least two generations a year.

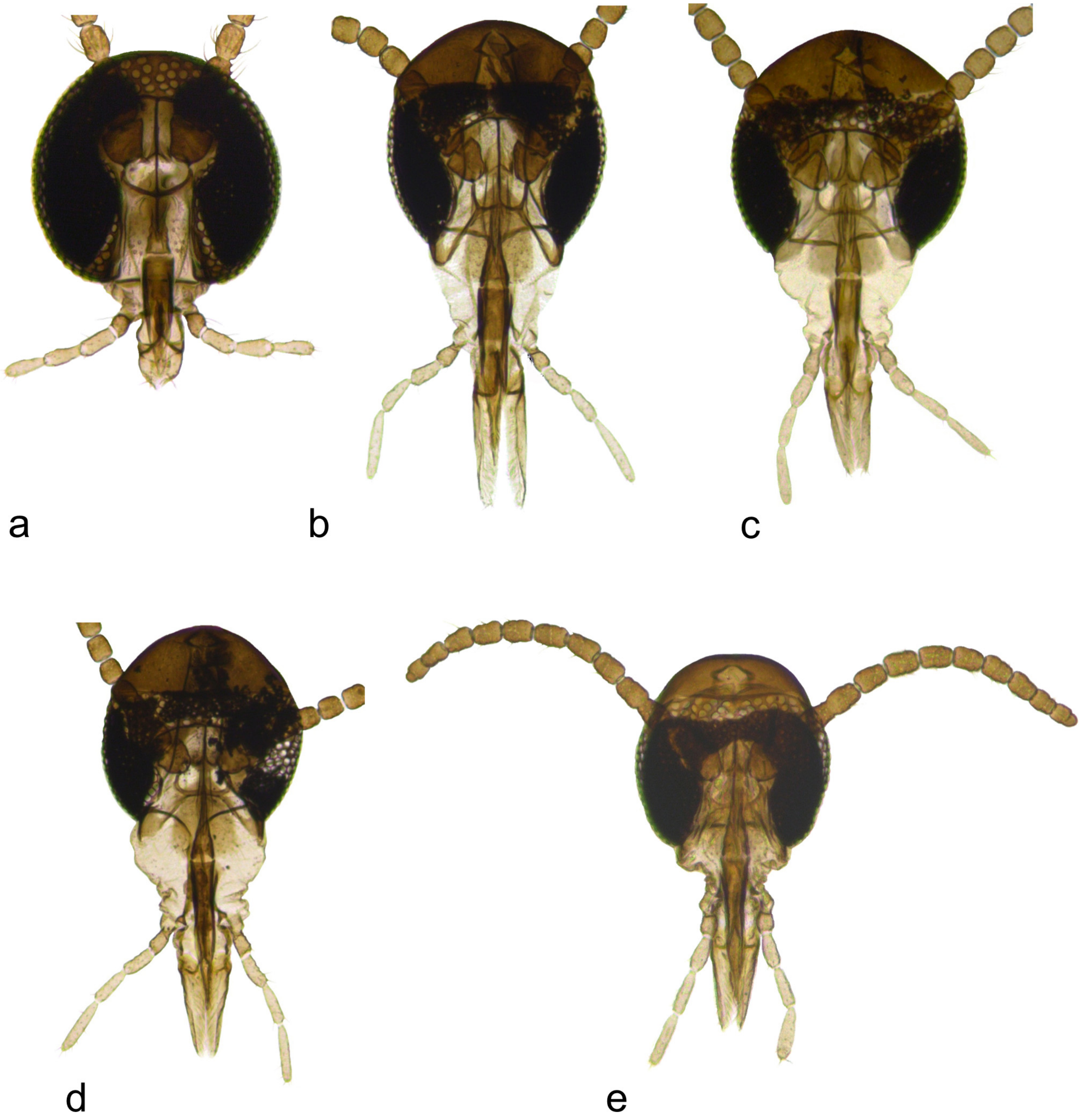

Fig 3. Adult heads. a. *Ozirhincus anthemidis*, male; b. *O*. *hungaricus*, male; c. *O*. *millefolii*, female; d. *O*. *longicollis*, female; e. *O*. *longicollis*, male, showing typical shape of flagellomeres.

doi:10.1371/journal.pone.0130981.g003

Key to the species of *Ozirhincus* Rondani

1. Proboscis short (Fig 3A): length, from base of labrum to tip of labella, shorter than height of eye. Occiput not encroaching eye area. Fourth palp segment about as long as third. On *Chrysanthemum* and *Anthemis* spp....................................*Ozirhincus anthemidis* (Rübsaamen)
   -. Proboscis long (Fig 3B–3E): length, from base of labrum to tip of labella, longer than

height of eye. Occiput encroaching eye area. Fourth palp segment usually notably longer than third. On various Anthemideae genera..2

2. Antennae with 11–12 flagellomeres. Pupal antennal horns with single pointed tip (Fig 4C and 4D). 3rd instar larva with 4 lateral and 3 terminal papillae on each side. On *Tanacetum* and *Tripleurospermum* spp.. *Ozirhincus hungaricus* Möhn

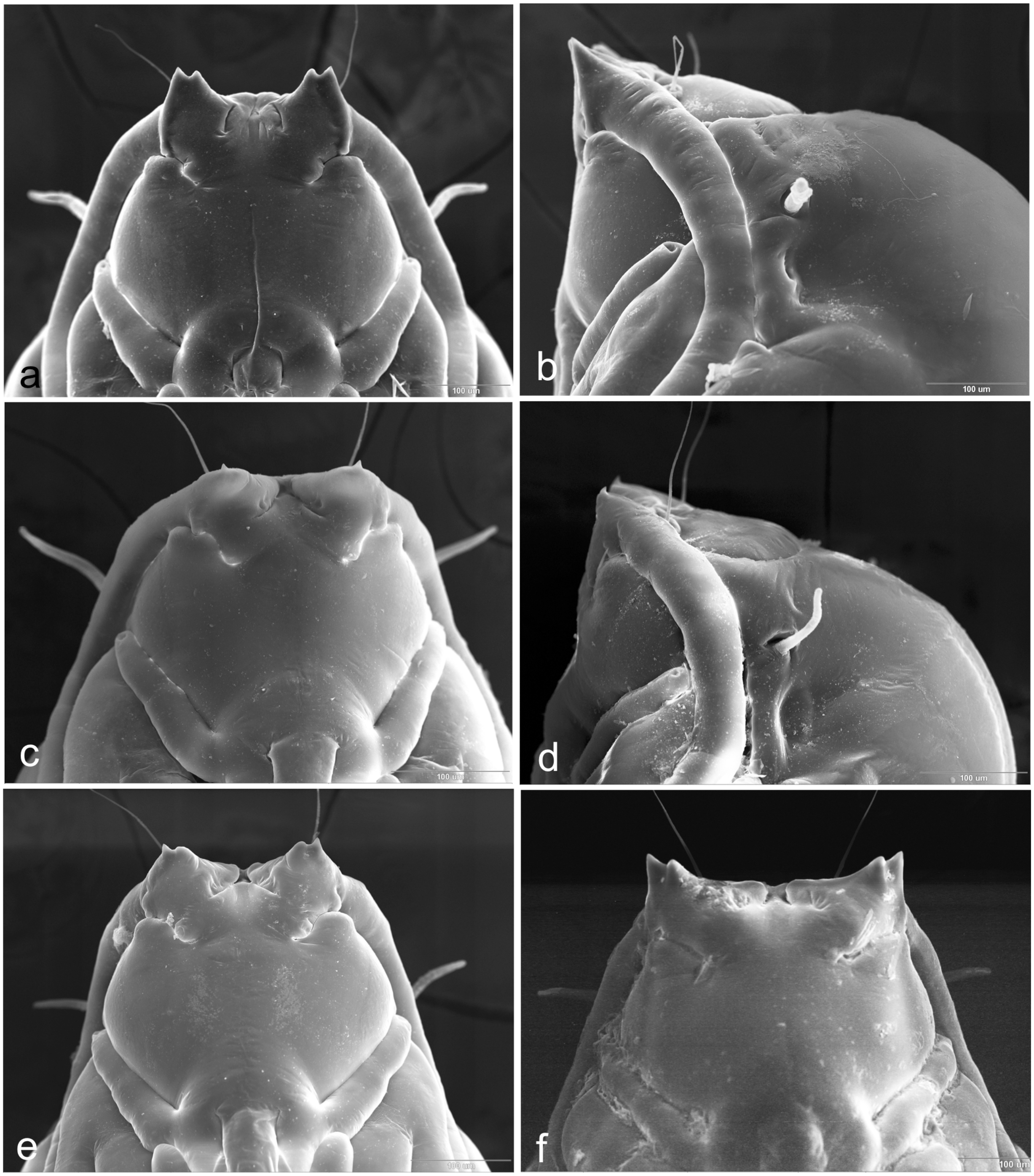

Fig 4. Pupae heads. a. *Ozirhincus anthemidis*, frontal; b. *O*. *anthemidis*, lateral; c. *O*. *hungaricus*, frontal; c. *O*. *hungaricus*, lateral; e. *O*. *longicollis*, frontal; f. *O*. *millefolii*, frontal.

doi:10.1371/journal.pone.0130981.g004

- Antennae with 8–10 flagellomeres. Pupal antennal horns bifid (Fig 4E and 4F). 3rd instar larva with 3 lateral and 2 terminal papillae on each side. On various Anthemideae genera. 3

3. Antennae with 8, occasionally 9 flagellomeres. Pupal antennal horns widely separated (Fig 4F). On *Achillea* spp.. *Ozirhincus millefolii* (Wachtl)
   - Antennae with 10, very rarely 9 flagellomeres. Pupal antennal horns not widely separated (Fig 4E). On *Tripleurospermum*, *Tanacetum*, *Leucanthemum* and *Anthemis* spp.. *Ozirhincus longicollis* Rondani

Descriptions

In the following descriptions, the names of host plants are given first because the identity of the host plant is one of the most important characters that may aid in species identification. Only those plant species that were confirmed as hosts in the present study are listed.

Ozirhincus anthemidis (Rübsaamen 1916)

Clinoryncha anthemidis Rübsaamen 1916: 561

Ozirhincus dalmaticus Möhn 1966

Ozirhincus kabylensis Möhn 1966

Host plants. *Chrysanthemum coronarium*, *C. segetum*, *Anthemis arvensis*, *A. bornmuelleri*, *A. rascheyana*, *A. retusa*, *A. tinctoria*.

Adult. ***Head*** (Fig 3A): Eye facets round; more spaciously arranged on vertex than laterally; eye bridge 3-4-facets long. Antenna (Fig 5A–5C): scape wide trapezoidal; pedicel globose; number of flagellomeres 11–12 in both sexes, rarely 10, number occasionally differs between antennae of same individual (n = 102♀, 104♂); flagellomeres globular to almost quadrate in female, more cylindrical in male (Fig 5B and 5C); first two flagellomeres usually partially to entirely fused, apical flagellomere often longer, evidently composed of 2–3 entirely or partially fused units; adjacent flagellomeres sometimes fused in mid antenna (Fig 5C); each flagellomere with two whorls of appressed circumfila and two rows of strong setae originating from prominent sockets, one row proximal to and one row between circumfila (Fig 5A); entire flagellomere surface other than neck covered by microtrichia. Palpus 4-segmented; segment 1 only slightly longer than wide, segments 3–4 about same length, 3 times longer than wide, with several strong setae and otherwise setulose. Frontoclypeal membrane with several strong setae on each side. Labrum about 4 times as long as width at base, parallel sided on basal two thirds, tapering from apical third towards setulose apex, with a few strong setae dorsally. Labella (Fig 6A) about 2.5 times as long as wide, somewhat concave medially, tapered apically, with several strong setae and densely setose along medio-apical surface.

Thorax: Dark grey, covered by white scales and setae. Anepimeron with group of 15–20 setae; other pleura without setae. Legs: dorsal part densely covered by black scales other than a patch of white scales on basal half of first tarsomere (Fig 2A–2C); ventral part densely covered by white scales. Tarsal claws (Fig 6C–6E) evenly curved, with thin tooth, strongly curved close to base; empodia longer than bend in claw; pulvilli about 0.3 times as long as claw. Wing: hyaline, with sparse delicate hairs on entire surface and long hairs along posterior margin; length 1.09–1.76 mm in males (n = 65), 1.09–1.66 mm in females (n = 58); R_{4+5} joins C around mid-length of wing, densely covered by mixed black and white scales; C with break after meeting point with R_{4+5}, densely covered by black scales almost to wing apex, except for patch of white scales at meeting point with R_{4+5} (Fig 2A–2C); M straight, CuA unforked. Stem of halter light orange, without scales; knob densely covered by black scales on basal third, white scales covering remainder.

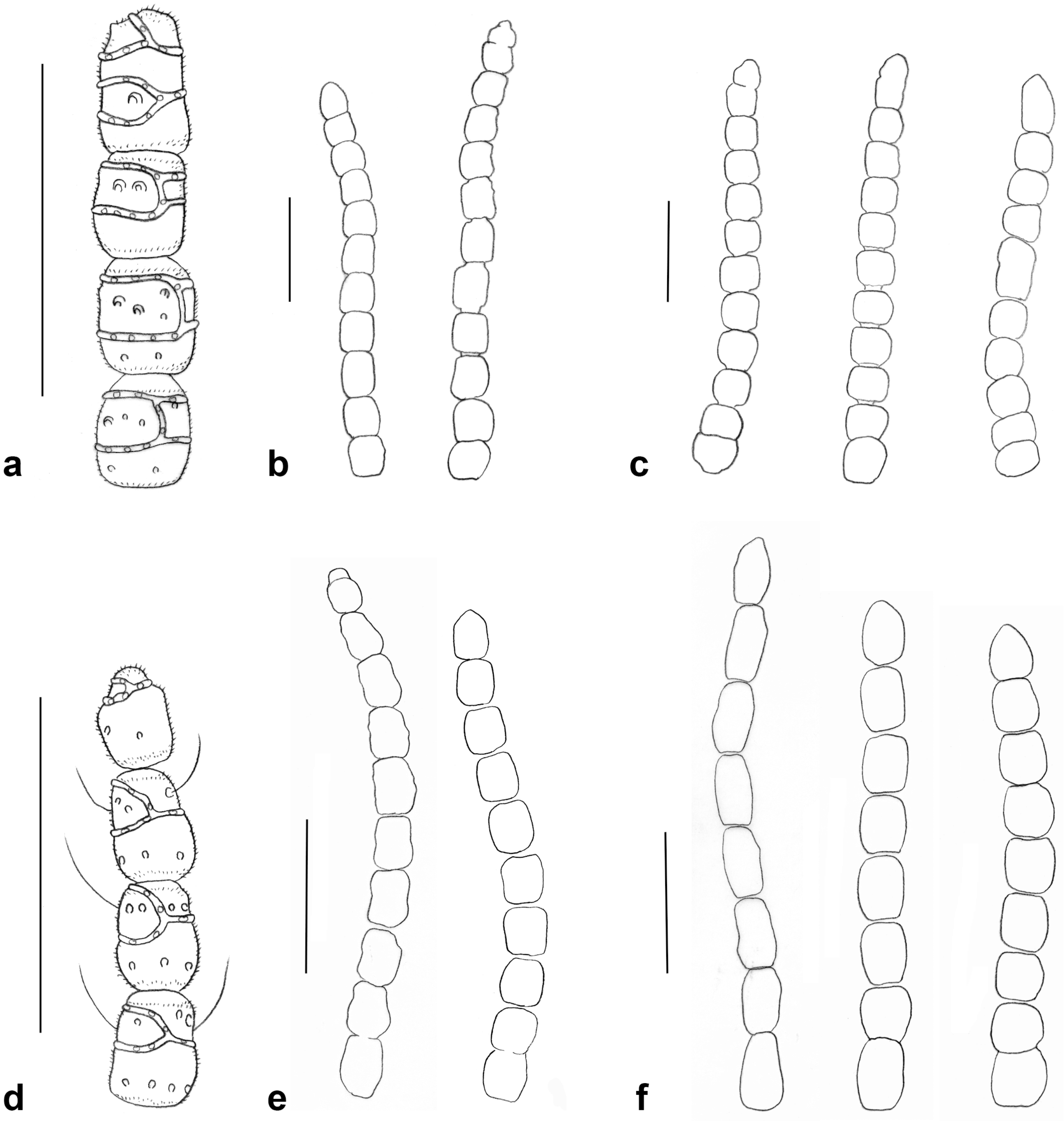

Fig 5. Antennae. a. *Ozirhincus anthemidis*, male apical flagellomeres, setae not shown; b. *O. anthemidis*, male flagellomeres, some setae shown; c. *O. anthemidis*, female flagellomeres; d. *O. longicollis*, male apical flagellomeres; e. *O. longicollis*, male (left), female (right); f. *O. millefolii*, male (left), female (center and right). Scale bars = 0.1 mm.

doi:10.1371/journal.pone.0130981.g005

Female abdomen (Fig 7A and 7B): Dorsum with dense covering of black and white scales: each tergite with wide transverse stripe of black scales on most of surface, and a narrow strip of

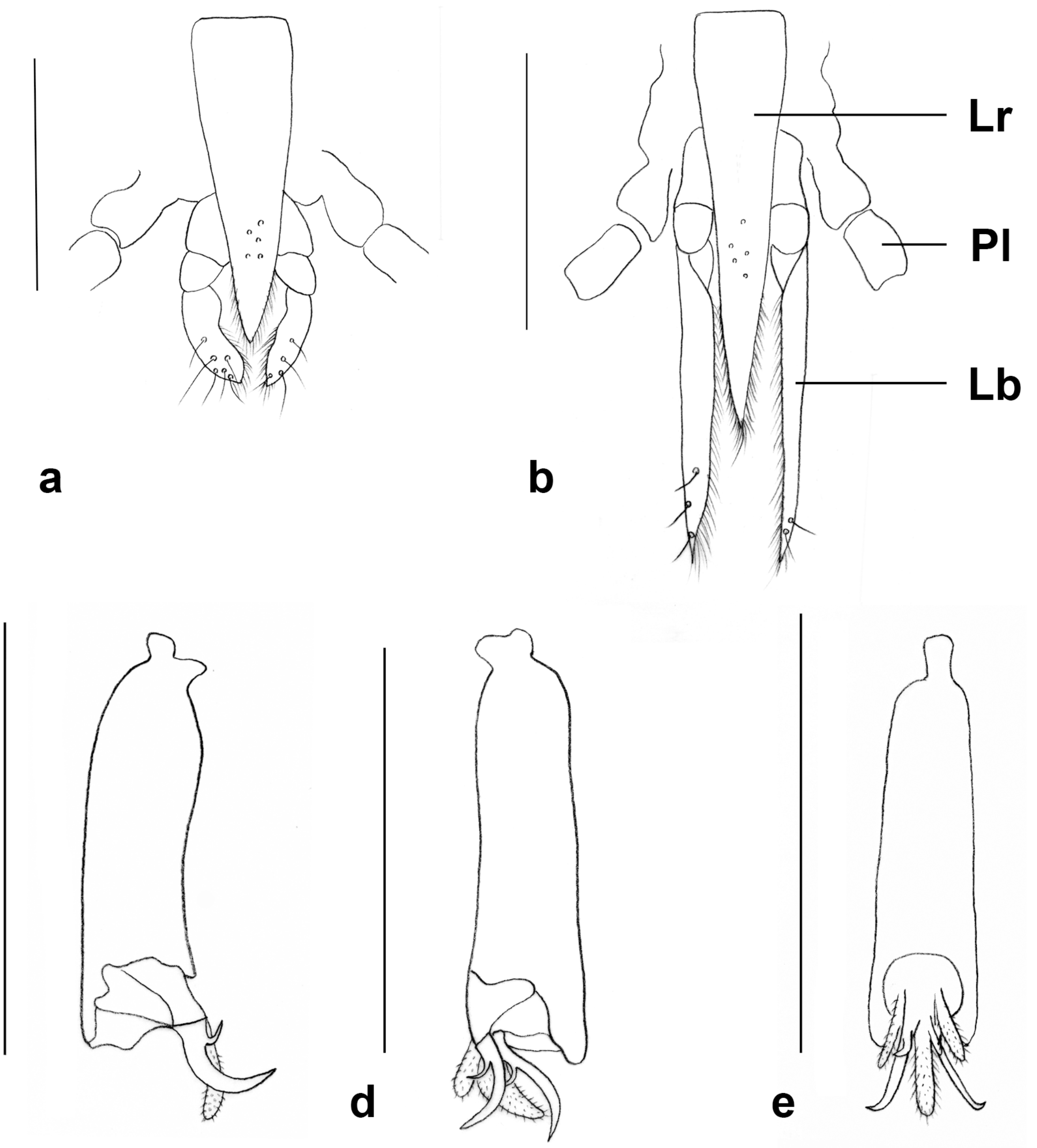

Fig 6. Proboscis. a. *Ozirhincus anthemidis*; b. *O. hungaricus*. c-e. *O. anthemidis*, fifth tarsomere, claw and acropod. c. Lateral; d. Lateral, showing both claws; e. Ventral. Lb–Labella, Lr–Labrum, Pl–Palp. Scale bars = 0.1 mm.

doi:10.1371/journal.pone.0130981.g006

white scales along posterior margin. Pleuron and venter with white scales. Tergites 1–6 rectangular, with anterior pair of trichoid sensilla, posterior row of strong setae, and otherwise evenly covered by scales; tergite 7 much smaller than preceding, pigmentation evanescent laterally at midlength, with anterior pair of trichoid sensilla and posterior row of setae; tergite 8 divided longitudinally into two elongate sclerites (Fig 7C), each with wide anterior area with pointed dorsal extension and wide posterior area with pointed ventral extension connected by long, narrow band; each sclerite with trichoid sensillum on anterior part of narrow band, and a group of strong, posterior setae on widened posterior area. Eighth tergite 1.65–2.77 times as long as seventh tergite (n = 56). Sternites 2–7 rectangular, with pair of closely approximated

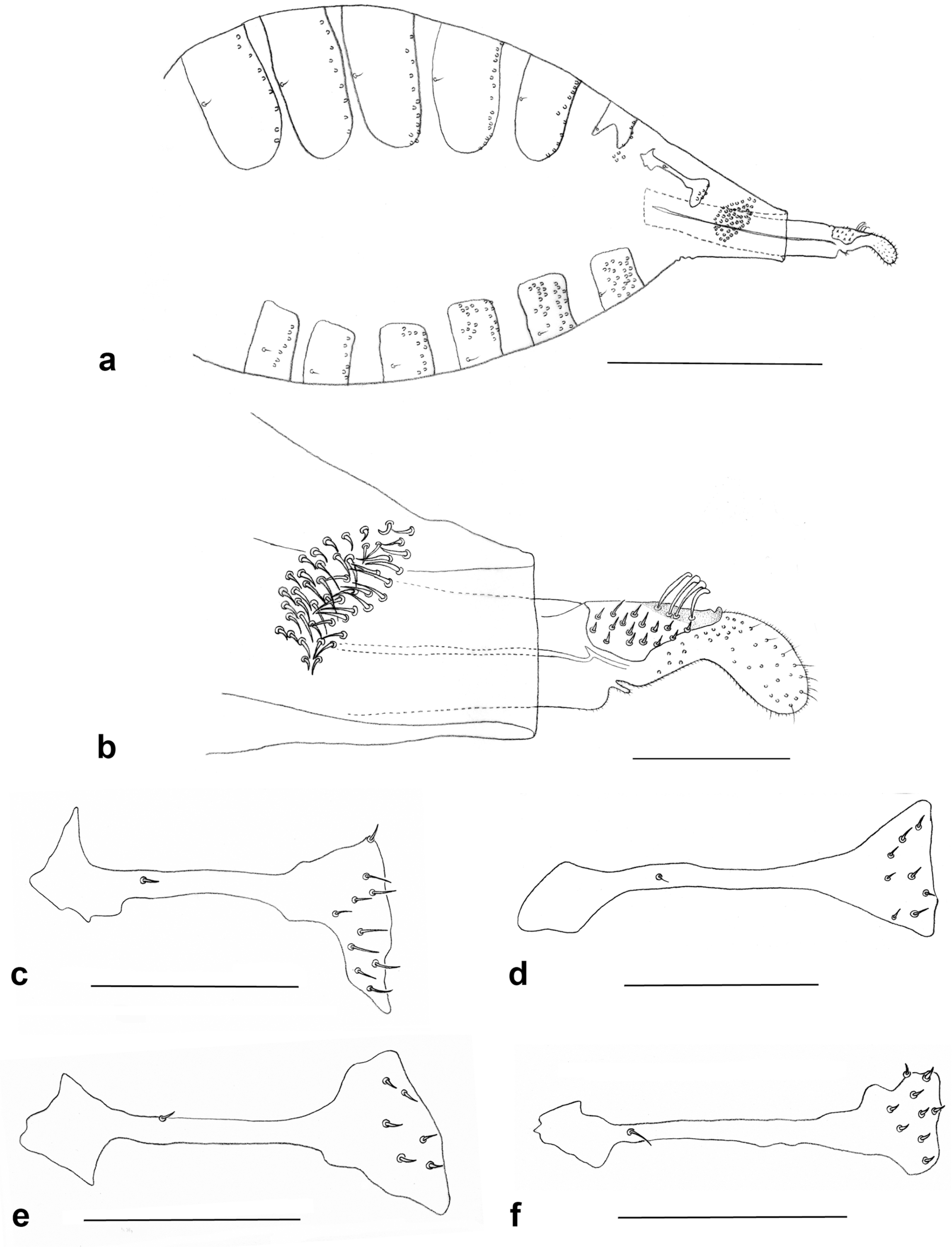
a
b
c
d
e
f

Fig 7. Female abdomen. a. *Ozirhincus anthemidis*, lateral; b. *O. anthemidis*, ovipositor, lateral; c. *O. anthemidis*, 7th tergite; d. *O. hungaricus*, 7th tergite; e. *O. longicollis*, 7th tergite; f. *O. millefolii*, 7th tergite. Al–Apical lamella, Dlp–Dorsolateral plate, Lgs–Lateral group of setae on eighth segment. Scale bars = 0.1 mm, except for Fig 28 = 0.5 mm.

doi:10.1371/journal.pone.0130981.g007

trichoid sensilla, posterior row of setae, and several setae laterally and medially, more numerous on more posterior segments; sternite 8 not apparent. Ovipositor long, protrosible, 3.57–7.47 times as long as eighth tergite (n = 57), with pigmented lateral sclerite along segment 9 and lateral group of strong, arched setae originating from prominent sockets, pointed mostly ventrally. Cercal segment (Fig 7B) with dorsolateral sclerotized plate more strongly pigmented along posterior area than elsewhere, laterally with 10–20 short, strong and straight setae; posterior pigmented area with small dorsal projection and bearing 3–4 very long, hook-like, blunt setae. Apical lamella cylindrical, evenly setulose, with numerous strong setae mostly concentrated on dorsal and apical areas. Hypoproct setulose.

Male abdomen (Fig 8A): Color pattern and scale covering as in female. Tergite 1 rectangular, with posterior row of strong setae, and evenly scattered scales; tergites 2–6 similar but larger and with anterior pair of trichoid sensilla; tergite 7 weakly sclerotized posteriorly, with fewer posterior setae, not forming a row; tergite 8 narrow, band-like, without setae other than anterior trichoid sensilla. Sternites 2–7 rectangular, with pair of closely approximated trichoid sensilla and 1–2 posterior rows of strong setae; posterior sternites with several strong setae medially, and otherwise evenly covered by scales; sternite 8 less pigmented and more setose than preceding but without trichoid sensilla. *Terminalia* (Fig 8B–8E): Gonocoxite cylindrical, about same width throughout length, with mediobasal lobe divided into prominent, globose, densely setose dorsal lobe, and elongate, ventral lobe tapering towards apex, and further subdivided into two longitudinal ridges on apical two thirds, sheathing aedeagus almost to apex (Fig 8D). Gonostylus widest at about mid length, tapering to wide comb-like tooth, with numerous setae, setulose on basal half dorsally and basal two thirds ventrally, remaining part with shallow ridges (Fig 8E). Aedeagus slightly longer than sheathing mediobasal lobes, wide and blunt apically. Hypoproct entire, blunt apically, or with slight, shallow notch, setose and setulose. Cerci separated by a deep notch, setose and setulose.

Larva (third instar) (Fig 9A–9C)**.** Light to dark yellowish-orange. Cylindrical-ovate. Integument covered by rounded verrucae. Antennae 1.5–2.0 times as long as wide. Cephalic apodeme considerably longer than head capsule (Fig 9A). Spatula (Fig 9A and 9H) long shafted and bidentate; shape of teeth and distance between them highly variable; when teeth farther apart, sometimes with minute additional projection between them. Sternal papillae without setae; pleural and dorsal papillae with long setae. On each side of spatula 3–4 asetose lateral papillae grouped together, and one asetose ventral papilla somewhat farther away (Fig 9B). Terminal abdominal segment with 2–3 setose papillae on each side (Fig 9C). All specimens obtained in the present study had 5 lateral papillae on each side; number of terminal papillae varied among individuals from different host plants: those from *Chrysanthemum* spp. and *Anthemis rascheyana* had 2 papillae on each side, those from *A. tinctoria* had 3, and those from *A. bornmuelleri* had either 2 or 3.

Pupa (Fig 4A and 4B)**.** Light to vivid orange. Antennal bases enlarged, forming straight bidentate horns, tapered and pointed ventroapically. Vertex with long and thin cephalic seta on each side, situated on conspicuous bulge. Face without apparent papillae. Prothoracic spiracle long and slender. Abdominal segments covered by acute spicules.

Distribution. Europe and circum-Mediterranean.

Material examined. The type series of *O. anthemidis* includes males, females, and pupal exuviae that have been kept in ethanol vials since their collection. This material was mounted

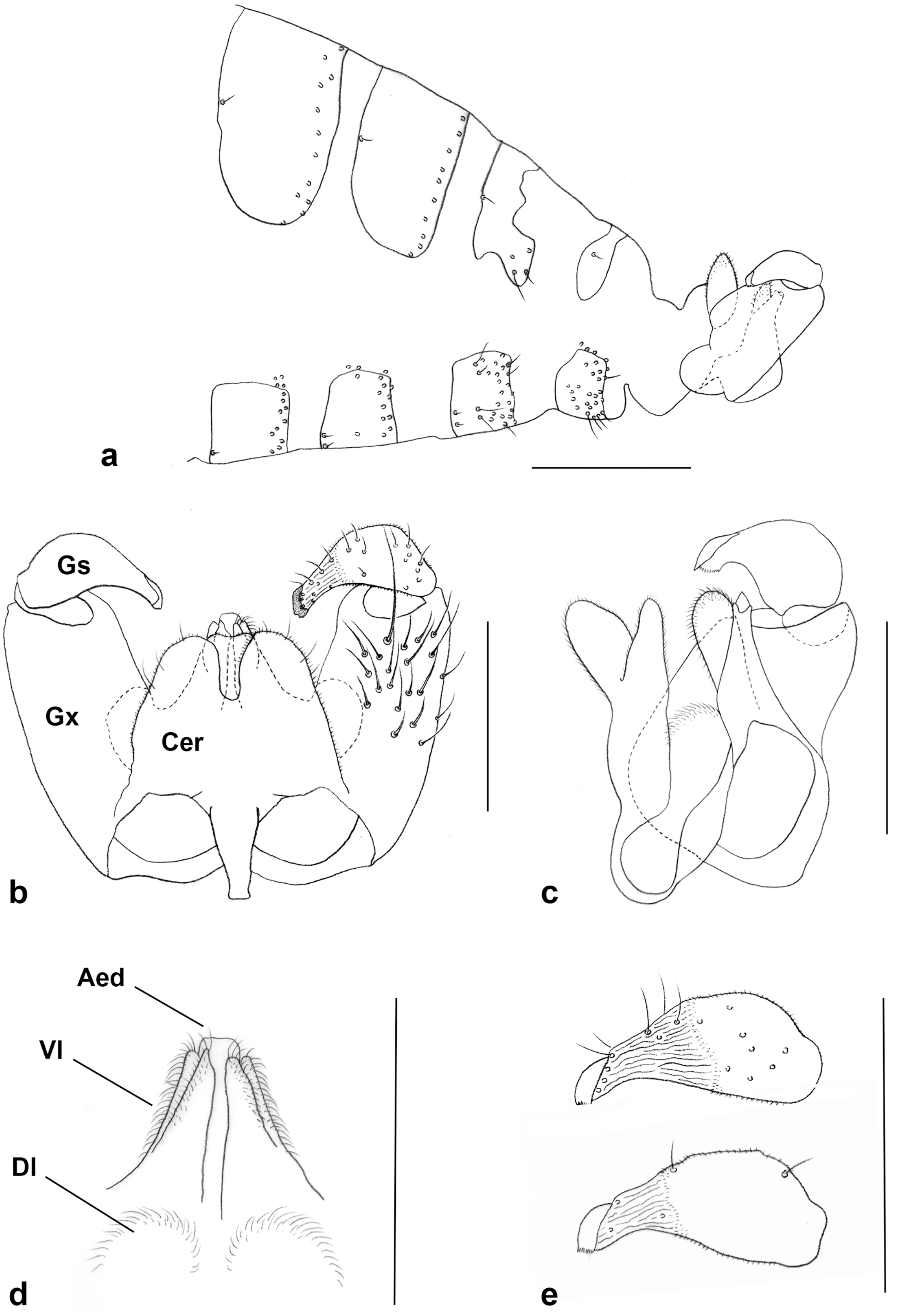
a
Gs
Gx
Cer
b
c
Aed
Vl
Dl
d
e

Fig 8. Male abdomen, *Ozirhincus anthemidis*. a. Post abdomen and terminalia, lateral; b. Terminalia, dorsal, setation shown on right gonopod; c. Terminalia, lateral, one gonopod removed; d. Terminalia, ventral, showing mediobasal lobes and aedeagus; e. Gonostylus, dorsal (top), ventral (bottom). Aed–Aedeagus, Cer–cercus, Dl–Dorsal part of mediobasal lobe, Gs–Gonostylus, Gx–Gonocoxite, Vl–Ventral part of mediobasal lobe. Scale bars = 0.1 mm.

doi:10.1371/journal.pone.0130981.g008

on permanent microscope slides in euparal for the purpose of the present study, and used for the designation of the following types: LECTOTYPE: ♀, Germany, Werlau, 1895, EH Rübsaamen, reared from *Anthemis tinctoria*, (115e). The lectotype is mounted on a permanent microscope slide in euparal, is in fair condition, and deposited in ZMHB. PARALECTOTYPES: 1♂, 2 exuviae, Germany, Werlau, 1895, EH Rübsaamen (115e) (ZMHB) (same data as lectotype; both exuviae on same slide); 2♀, 2♂, 4 exuviae, Germany, Oberheimbach, 17.viii.1906, EH Rübsaamen (115b) (all exuviae on same slide) (ZMHB); 3♀, 3♂, Germany, no locality or date given, EH Rübsaamen (115a) (ZMHB).

OTHER MATERIAL EXAMINED: ex *Chrysanthemum coronarium*: 3♀, 2♂, Israel, Herzeliya, 8.v.2009, A. Freidberg; 27♀, 20♂, 14 larvae, Israel, Herzeliya, 19.iv.2012, A. Freidberg (2♀, 2♂ ZMHB, 2♀, 2♂ SMNS, 1♀, 1♂ ZFMK, others TAUI); 4♀, 3♂, Israel, Kefar Hahoresh, 28.iv.2012, N. Dorchin; 4♀, 4♂ (on slides), 12♀, 12♂ (pinned), Israel, Hadera, 11.iii.2013, N. Dorchin and I. Hayon; 1♀, 6♂, Israel, Ziqim, 4.iv.2013, N. Dorchin.

Ex *Chrysanthemum segetum*: 4♀, 11♂, Isarel, Dan, 10.iv.2014, N. Dorchin.

Ex *Anthemis bornmuelleri*: 5♂, 13 larvae, Israel, Ma'agar Bental, 14.v.2012, N. Dorchin; 19♀, 21♂, 7 larvae, Israel, Ma'agar Bental, 25.v.2012, N. Dorchin (2♀, 2♂ ZMHB, others TAUI); 3♂, Israel, Ma'agar Bental, 12.v.2014, N. Dorchin.

Ex *Anthemis rascheyana*: 1♀, 4♂, 5 larvae, Israel, Mt. Hermon, 1750m, 24.v.2012, N. Dorchin; 8♀, 7♂, 6 larvae, Israel, Mt. Hermon, 1780m, 6.vi.2012, N. Dorchin.

Ex *Anthemis retusa*: 9♀, 4♂, 5 larvae, Israel, Nahal Nizzana, 12.v.2013, N. Dorchin.

Ex. *Anthemis tinctoria*: 5♀, Israel, Newe Ativ, 15.v.2012, N. Dorchin; 5♀, 6♂, Israel, Newe Ativ, 25.v.2012, N. Dorchin; 8♀, 2♂, 12 larvae, Israel, Newe Ativ, 6.vi.2012, N. Dorchin.

Biology. The phenology of *O. anthemidis* varies within its distribution range and in relation to its host plants. In dry Mediterranean regions (Middle East and North Africa), the main activity period is in spring, whereas in continental Europe adults are active also in summer. Adults of the overwintered generation emerge in Europe from mid-March to late April and those of the subsequent generation between August and October [11]. In Israel, adults associated with *Chrysanthemum coronarium* emerge in March from infested achenes in the ground from the previous year, and can be seen hovering over and standing on developing inflorescences, where females lay their eggs into individual flowers. Larvae develop and soon pupate inside the achenes, preventing them from developing seeds. Adults of the subsequent generation emerge already in April, in contrast to the much slower development of larvae in Europe. Adults reared in May may represent either late-developing individuals of the second generation or those of a third generation. Infested achenes are slightly inflated and enlarged compared to normal achenes, and the larvae or pupae fill them completely making it hard to dissect them from the achenes without damaging them. Females of the last generation lay eggs in the flowers, and the hatching larvae develop to third instars but do not pupate. Instead, they enter a diapause inside the achenes that drop to the ground when the plants have dried up, and will emerge as adults the following year. The life history of populations associated with *Anthemis* spp. in Israel is essentially similar except that the main activity period is in late rather than early spring. Diapause takes place in the summer through winter months in Mediterranean regions, or only during the autumn and winter in continental Europe.

Remarks. Rübsaamen [9] repeated Loew's [26] rearings from *Tripleurospermum inodorum* and *Anthemis arvensis*, as well as from *A. tinctoria*, but noticed that he was getting two

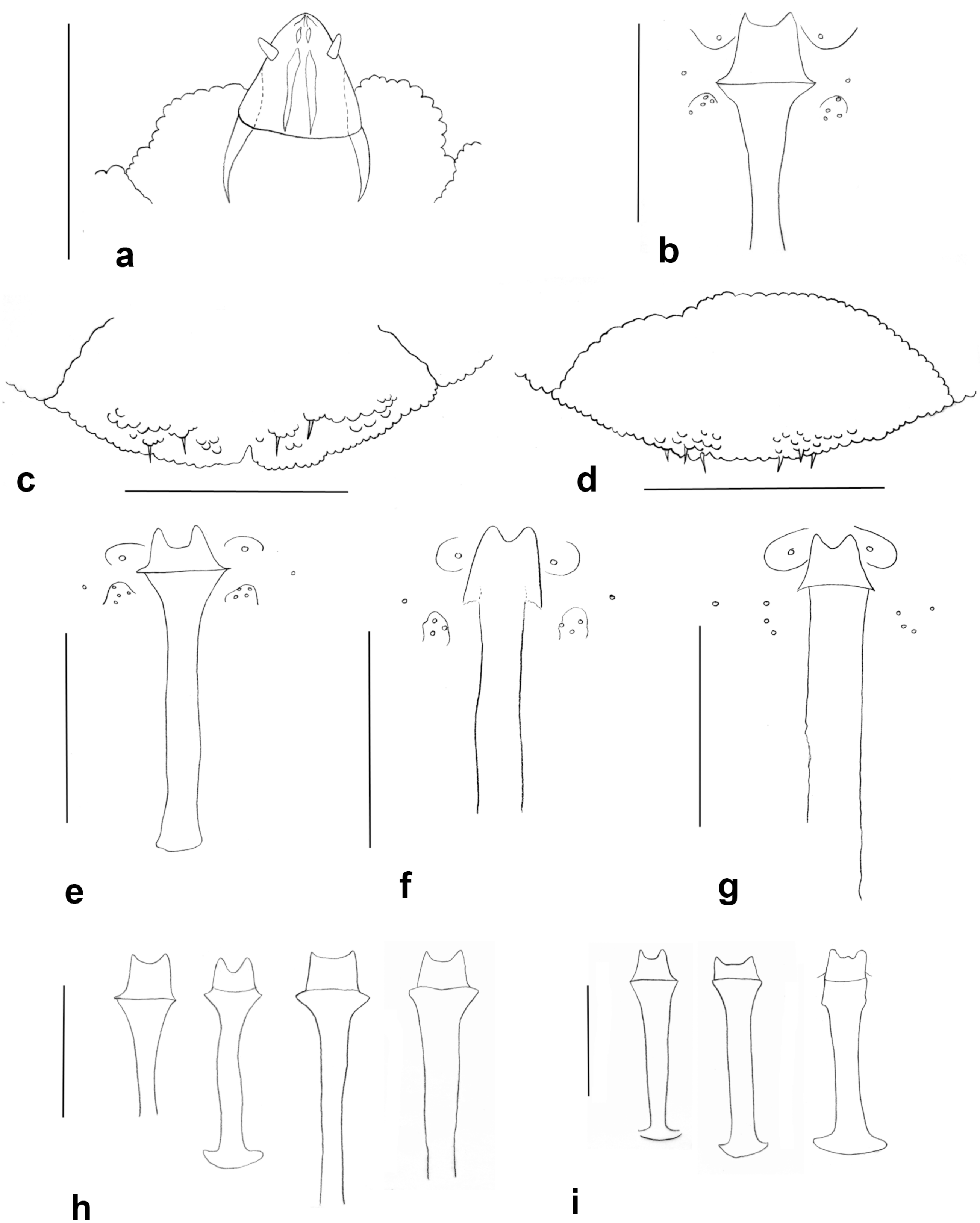

Fig 9. Larvae. a. *O. anthemidis*, head; b. *O. anthemidis*, spatula and associated papillae; c. *O. anthemidis*, terminal abdominal segment; d. *O. hungaricus*, terminal abdominal segment. e. Spatula and associated papillae, *O. hungaricus*; f. Spatula and associated papillae, *O. longicollis*; g. Spatula and associated

papillae, *O. millefolii*; h. Variation of spatula shape in *O. anthemidis*; i. Variation of spatula shape in *O. hungaricus*. Lp–Lateral papillae, Sp–Sternal papilla, Vp–Ventral papilla. Scale bars = 0.1 mm.

doi:10.1371/journal.pone.0130981.g009

types of adults from the latter two hosts: some with a rather short proboscis and some with the typical long proboscis mentioned by Loew for his *Clinorhyncha chrysanthemi* (currently *Ozirhincus longicollis*). Rübsaamen wondered how Loew had not noticed the two different populations, and speculated that he did not manage to rear the short-snouted individuals for some reason. He correctly attributed the long-snouted adults to *Clinorhyncha chrysanthemi*, noting that they had 10 antennal flagellomeres as opposed to 11–12 in the short-snouted specimens, and described the latter as a new species–*Clinorhyncha anthemidis* (currently *Ozirhincus anthemidis*).

Ozirhincus anthemidis is easily distinguishable from all other species in the genus by its short proboscis, short fourth palpal segment, and 11–12 antennal flagellomeres, as opposed to 8–9 in *O. millefolii* and 10 in *O. longicollis*. *Ozirhincus hungaricus*, the only other species in the genus with a regular number of 11–12 antennal flagellomeres, has a much longer proboscis and is found on different host plants.

Ozirhincus anthemidis is a very common species throughout Europe and the Mediterranean region. It was previously known only from *Anthemis* hosts [12], [13], but in the present study we found that it is also very common on *Chrysanthemum coronarium* and *C. segetum* (and it was the only species we reared from those plants). Host plants that were reported for *O. anthemidis* by Möhn [12] but not verified in the present study are *Anthemis austriaca*, *A. cretica* (as *A. montana*), *A. kotschyana*, and *A. cotula*. *Anthemis arvensis* is listed here as a verified host plant based on the information in Rübsaamen's original description of *O. anthemidis*.

Ozirhincus dalmaticus and *O. kabylensis* were described by Möhn [12] as belonging to the '*anthemidis* group' based on small differences in the larval spatula and chaetotaxy, but were later synonymized under *O. anthemidis* by Skuhravá [13], with whom we concur.

Ozirhincus hungaricus Möhn 1968

Ozirhincus hungaricus Möhn 1968: 55

Ozirhincus hispanicus Möhn 1968

Ozirhincus parvus Möhn 1968

Characters as in *O. anthemidis* except for the following:

Host plants. *Tanacetum vulgare*, *Tripleurospermum inodorum*, and *Tanacetum corymbosum*.

Adult. ***Head*** (Fig 3B): Occiput extending forward at expense of eye area in frontal view; eye bridge about 4 facets long. Antenna: number of flagellomeres 11–12 in both sexes (n = 100♀, 89♂); flagellomeres short-cylindrical to almost quadrate in female, more elongate in male; first two flagellomeres usually partially to entirely fused, apical flagellomere slightly tapered, sometimes appear as if 'budding' to form the beginning of a small additional segment. Palpus: fourth segment 1.3–2.0 times longer than third. Frontoclypeal membrane greatly extended, with many strong setae on each side. Labrum long, triangular: at least 4 times as long as width at base, tapering and strongly setose on apical quarter. Labella more than 10 times as long as wide, somewhat concave medially, tapered apically, with several strong setae towards apex and densely setose along entire medial surface (Fig 6B).

Thorax: Anepimeron with a group of 10–20 setae. Wing length: 1.34–1.73 mm in males (n = 38), 1.28–1.67 mm in females (n = 47).

Female abdomen: Elongate sclerites of tergite 8 (Fig 7D) each with wide anterior area, usually with rounded rather than pointed dorsal and ventral extensions and wide posterior area connected by long, narrow band; each sclerite with trichoid sensillum on anterior third of

narrow band, and 8–10 posterior setae on widened posterior area. Eighth tergite 1.64–3.34 times as long as seventh tergite (n = 46). Ovipositor 4.13–7.77 times as long as eighth tergite (n = 46).

Larva. (third instar). Integument covered by rounded verrucae. Antennae 2–3 times as long as wide. Cephalic apodeme considerably longer than head capsule. Spatula (Fig 9E and 9I) long-shafted and bidentate; teeth widely separated by concave to straight gap, sometimes with minute additional projection between them. Sternal papillae without setae; pleural and dorsal papillae with long setae; on each side of spatula 4 asetose lateral papillae grouped together, and one asetose ventral papilla somewhat farther away (Fig 9E). Eighth abdominal segment with 3 setose papillae on each side (Fig 9D).

Pupa (Fig 4C and 4D)**.** Antennal bases enlarged, bulging, with tiny tapered tip at center of bulge.

Distribution. Widespread in Europe to Siberia.

Material examined. HOLOTYPE: Larva, Hungary, Ménesi, Nagyboldogasszony-útja, 21. viii.1949, dissected by E. Möhn from *Tanacetum corymbosum*. The holotype is the left specimen of two larvae mounted under the same cover glass on a permanent microscope slide in euparal, and is deposited in the SMNS. The second larva on the same slide is currently labeled as a paratype.

PARATYPE: 1 larva, same data as holotype (SMNS).

OTHER MATERIAL EXAMINED: ex *Tanacetum vulgare*: 1♀, 1♂, Czech Republic, Moravia, Břeclav, 1.ix.1958, M. Skuhravá (Skuhravá collection, as *O. tanaceti*); 3♀, 2♂, Hungary, Szentendre, 5.viii.1988, M. Skuhravá (in ethanol); 6♀, 8♂(in ethanol), UK, London, vi.2005, B. Wurzell; 12♀, 9♂ (on slides), 1♀, 1♂ (in ethanol), Germany, NRW, Ägidienberg, 11.viii. 2011, N. Dorchin; 25♀, 21♂, 13 larvae (on slides), 11♀, 14♂ (in ethanol), 4♀ (pinned), Germany, NRW, Wahner Heide, 11.viii. 2011, N. Dorchin, (1♀, 1♂ on slides ZFMK, others TAUI); 12♀, 11♂ (on slides), 6♀, 10♂ (in ethanol), 6♀ (pinned), Germany, NRW, Leverkusen, 14.viii. 2011, E. Diehl; 5♀, 4♂, the Netherlands, Ede, central station, 25.viii.2011, E. Dijsktra (in ethanol).

Ex *Tripleurospermum inodorum*: 1♀, 1♂, UK, Surrey, Send, Woodhill, 20.viii.2011, KM Harris and N. Dorchin; 23♀, 11♂, 12 larvae, Germany, Leverkusen, 30.viii.2011, E. Diehl (1♀, 1♂ ZFMK, 1♀, 1♂ ZMBH, others TAUI).

Biology. The life history of this species is similar to that of *O. longicollis*. An ovipositing female and infested achenes of the main host plant, *Tanacetum vulgare*, are shown in Fig 2A–2D.

Remarks. Möhn [12] described *O. hungaricus*, *O. parvus*, and *O. hispanicus* as belonging to the 'tanaceti group' based on characters of the larvae alone, but these species were later synonymized under *O. tanaceti* by Skuhravá [13]. The larvae of all species in the 'tanaceti group' have 4 lateral papillae on each side of the spatula and 3 terminal papillae on each side of the anus. Because the holotype larva of *Ozirhincus hungaricus* survived in a recognizable condition while those of *O. parvus* and *O. hispanicus* did not, we reinstate *O. hungaricus* as the name of the common species associated with *Tanacetum vulgare* in Europe, and synonymize the other two species under it. *Tanacetum corymbosum* is listed here as a confirmed host because the holotype of *O. hungaricus* had been found in that plant [12].

In the present study we found that, in addition to *T. vulgare*, *O. hungaricus* also develops in *Tripleurospermum inodorum*, which it sometimes shares with *O. longicollis*. When the two species occur together on that host plant, they can be distinguished from each other easily by: the number of antennal flagellomeres (10 in *O. longicollis* vs. 11–12 in *O. hungaricus*); the characteristic constrictions of the male flagellomeres in *O. longicollis*, where they are girdled by the circumfila loops (Figs 3E, 5D and 5E); the clear difference in the shape of the pupal antennal horns (Fig 4E vs. Fig 4C); and by the following combination of characters in the third-instar

larva: the teeth of the spatula in *O. longicollis* are usually closer together than in *O. hungaricus* (compare Fig 9F and 9E); *O. longicollis* has 3 lateral papillae on each side of the spatula and 2 terminal papillae on each side of the anus, as opposed to 4 lateral papillae and 3 terminal papillae on each side in *O. hungaricus*.

Ozirhincus longicollis Rondani 1840

Ozirhincus longicollis Rondani 1840: 16

Clinorhyncha chrysanthemi Loew 1850

Clinorrhyncha crassipes Winnertz 1853

Clinorrhyncha tanaceti Kieffer 1889 –**new synonym**

Clinorrhyncha leucanthemi Kieffer 1898

Characters as in *O. anthemidis* except for the following:

Host plants. *Tripleurospermum inodorum*, *Anthemis arvensis*, *A. bornmuelleri*, *A. cotula*, *A. pseudocotula*, *A. rascheyana*, *Leucanthemum vulgare*, *Tanacetum balsamita*, *T. coccineum*, *T. parthenium*, *T. poteriifolium*, *T. vulgare*.

Adult. ***Head*** (Fig 3D and 3E): Occiput extending forward at expense of eye area in frontal view; eye bridge about 4 facets long. Antenna: number of flagellomeres 10 in both sexes (n = 57♀, 36♂); flagellomeres short-cylindrical to almost quadrate in female, more elongate in male (Fig 5E); first two flagellomeres usually partially to entirely fused, apical flagellomere slightly tapered, sometimes appears as if 'budding' to form the beginning of a small additional segment; male flagellomeres characteristically constricted in mid-section by circumfila loops (Figs 3E and 5D). Palpus: fourth segment usually 1.3–1.6 times longer than third. Frontoclypeal membrane greatly extended, with many strong setae on each side. Labrum long-triangular: at least 4 times as long as width at base, tapering and strongly setose on apical quarter. Labella more than 10 times as long as wide, somewhat concave medially, tapered apically, with several strong setae towards apex and densely setose along entire medial surface.

Thorax: Anepimeron with a group of 5–15 setae. Wing length: 1.13–1.57 mm in males (n = 19), 1.18–1.64 mm in females (n = 22).

Female abdomen: Elongate sclerites of tergite 8 (Fig 7E), each with wide anterior area with pointed dorsal and ventral extensions and wide posterior area connected by long, narrow band; each sclerite with trichoid sensillum on anterior part of narrow band, and 4–6 posterior setae on widened posterior area. Eighth tergite 1.53–2.51 times as long as seventh tergite (n = 22). Ovipositor 3.90–7.24 times as long as eighth tergite (n = 12).

Larva. (third instar). Integument covered by rounded verrucae. Antennae 2–3 times as long as wide. Cephalic apodeme considerably longer than head capsule. Spatula (Fig 9F) long shafted and bidentate; teeth separated by narrow gap. On each side of spatula 3 asetose lateral papillae grouped together, and one asetose ventral papilla somewhat farther away. Eighth abdominal segment with 2 setose papillae on each side.

Pupa (Fig 4E)**.** Light to vivid orange. Antennal bases enlarged, tapered into short bi-dentate horns; lateral lobe tapered, median lobe rounded.

Distribution. Europe, Israel. Probably circum-Mediterranean.

Material examined. The type of *Ozirhincus longicollis* Rondani is considered lost, based on an exhaustive study of Rondani's collection by Gagné and Solinas [27]. Given that this is the type species of *Ozirhincus*, we hereby designate a neotype for it in order to clarify the application of the name *O. longicollis* Rondani and the generic concept of *Ozirhincus* as a whole. Although the host plant from which *O. longicollis* was described in Italy is unknown, the morphological description given by Rondani is distinctive, and the species has been reported since from various locations and host plants throughout Europe [9–11], [26]. Based on this information, we designate the neotype from Germany, from the first host plant with which this species has been associated [26].

NEOTYPE: ♀, Germany, Leverkusen, 30.viii.2011, E. Diehl, reared from *Tripleurospermum inodorum*. The neotype is mounted on a permanent microscope slide in euparal and deposited in TAUI.

OTHER MATERIAL EXAMINED: 1♀, UK, Surrey, Wisley, 19.vi.1926, HF Barnes, ex *Leucanthemum vulgare* (as 'Oxeye Daisy'), BMNH(E) 1633265; 9♀, 2♂, UK, RHS Wisley, 13.vii.1953, HF Barnes, ex. *Tanacetum coccineum* (as *Chrysanthemum coccineum*), *Tanacetum parthenium* (as *Chrysanthemum parthenium*), and *Tanacetum poteriifolium* (as *Chrysanthmum cassium*), BMNH(E) 1633190, 1633192, 1633228–9, 1633232–5, 1633240–1, 1633243–4; 1♀, UK, Lincolnshire, Barnack station, near Stamford, HF Barnes, ex *Leucanthemum vulgare* (as 'wild Oxeye Daisy'), BMNH(E) 1633239; 13♀, 2♂, UK, Hertsfordshire, Bayfordbury, HF Barnes, ex *Tanacetum poteriifolium* (as *Chrysanthemum cassium*), *Tanacetum balsamita* (as *Chrysanthemum balsamita*), and '*Chrysanthemum* sp.' (most probably *Tanacetum* sp.), BMNH(E) 16331989, 1633-201-7, 1633224–5, 1633242, 1633248, 1633249–251; 1♂, Czech Republic, Bohemia, Dolni Poćenice, 13.viii.1956, M. Skuhravá, ex *Anthemis arvensis* (Skuhravá collection, as *O. anthemidis*); 1♀, 1♂, Czech Republic, Bohemia, Davle, 7.vii.1958, M. Skuhravá, ex *Anthemis arvensis* (Skuhravá collection, as *O. anthemidis*); 3♀, 2♂, Czech Republic, Petrovice, 22.vii.1964, M. Skuhravá, ex *Anthemis cotula* (in ethanol); 1♀, Czech Republic, Bohemia, ŹameÆl, 16.viii.1964, M. Skuhravá, ex *Tripleurospermum inodorum* (as *Matricaria inodora*) (Skuhravá collection, as *O. anthemidis*); 2♀, Czech Republic, Bohemia, ZámeÆl, 7.v.1965, M. Skuhravá, ex *Leucanthemum vulgare* (in ethanol); 2♀, 2♂, Czech Republic, Rybná, 11.v.1965, M. Skuhravá, ex *Leucanthemum vulgare* (in ethanol); 2♀, 3♂, 2 larvae, Germany, Leverkusen, 30.viii.2011, E. Diehl, ex *Tripleurospermum inodorum* (same data as neotype); 4♀, 4♂, UK, Surrey, Send, Woodhill, 20.viii.2011, KM. Harris and N. Dorchin, ex *Tripleurospermum inodorum*; 11♀, 11♂, 4 larvae, Israel, Ma'agar Bental, 14.v.2012, N. Dorchin, ex *Anthemis bornmuelleri* (1♀,1♂ ZMBH, 1♀, 1♂ SMNS, 1♀, 1♂ NHMW, 1♀, 1♂ ZFMK, others TAUI); 1 larva, Israel, Mt. Hermon, 1750m, 24.v.2012, N. Dorchin, ex *Anthemis rascheyana*; 2♀, 1♂, Israel, Ma'agar Bental, 25.v.2012, N. Dorchin, ex *Anthemis bornmuelleri*; 2♀, Israel, Hermon, 1780m, 6.vi.2012, N. Dorchin, ex *Anthemis rascheyana*; 3♀, 7♂, Israel, Ma'agar Bental, 22.v.2014, A. Freidberg, ex *Anthemis bornmuelleri*; 6♀, 6♂, Israel, En Timrat 0.5km S, 4.iv.2015, N. Dorchin and U. Dorchin, ex *Anthemis pseudocotula* (in enthanol).

Biology. The life history of this species is similar to that of *O. anthemidis*. In the present study it was most abundant on *Tripleurospermum inodorum* and *Anthemis bornmuelleri*, from which adults emerged at the same time as those of *O. hungaricus* and *O. anthemidis*, respectively. The association of *O. longicollis* with the several *Tanacetum* spp. that are mentioned above is based on examination of material reared by HF Barnes [10] and housed at the BMNH, material received from the private collection of E. Dijkstra, and on Kieffer's original description of *O. tanaceti*.

Remarks. Rondani [7] described *Ozirhincus longicollis* without a host plant association, but mentioned that the proboscis is long, tapered, and held perpendicular to the head. He named the genus after this character, and the species after the long neck. Loew [26] later described *Clinorhyncha chrysanthemi*, also noting that the proboscis is very long and is bent under the head against the thorax, but he did not give a reason for its separation from *O. longicollis*. The two species were later synonymized by Möhn [11].

Two other species synonymized by Möhn [11] under *O. longicollis* in the same work are *Clinorrhyncha leucanthemi* and *C. crassipes*. The types of these species are lost, but information in subsequent publications [26], [9], [10], as well as our own findings support Möhn's synonymy. Winnertz [28] described *C. crassipes* without an associated host but mentioned that it had a long proboscis and 10 antennal flagellomeres in the male. This description places it clearly in *O. longicollis*. Kieffer [29] described *C. leucanthemi* from *Leucanthemum vulgare* (as

Chrysanthemum leucanthemum) without any further information, but detailed information was given in Barnes et al. [10], who reared it from the same host plant. Barnes et al. show the long proboscis and long fourth segment of the palp, and state that the species has 10 antennal flagellomeres. Again, this combination of characters fits only with *O. longicollis*.

Finally, *O. tanaceti*, described by Kieffer [30] from *Tanacetum vulgare*, is also synonymized here under *O. longicollis*. As in the above-mentioned cases, the type series of *O. tanaceti* is considered lost, hence our decision is based on Kieffer's original description and our own findings. In that description, Kieffer emphasized the fact that all 14 specimens he examined had 10 antennal flagellomeres, a character that fits only *O. longicollis*, based on our examination of 189 specimens from *T. vulgare* from the UK, Germany, Austria, Czech Republic, and the Netherlands. A single female we reared from *T. vulgare*, which had 10 antennal flagellomeres, was found to be *O. longicollis* based on DNA sequencing, corroborating our conclusion that Kieffer's specimens, upon which he based the description of *O. tanaceti*, actually constituted a series of *O. longicollis* individuals. This finding also confirms our conclusion that flagellomere number is a reliable character in *Ozirhincus*.

In the present study we found that *O. longicollis* often occurs together with *O. hungaricus* on *Tripleurospermum inodorum*. In this scenario, one can tell the two species apart by the differences described above under *O. hungaricus*. When *O. longicollis* occurs in the same host plants with *O. anthemidis*, the two species can be distinguished easily by their adult and pupal characters as described above under *O. anthemidis*.

Ozirhincus millefolii (Wachtl 1884)

Clinorrhyncha millefolii Wachtl 1884: 161

Clinorhyncha filicis Felt 1907

Clinorhyncha karnerensis Felt 1908

Characters as in *O. anthemidis* except for the following:

Host plants. *Achillea millefolium*, *Achillea ptarmica*.

Adult. ***Head*** (Fig 3C): Occiput extending forward at expense of eye area in frontal view; eye bridge 2-facets long. Palpus: segment four usually 1.2–1.7 times longer than segment three. Frontoclypeal membrane greatly extended, with many strong setae on each side. Labrum long triangular: at least 4 times as long as width at base, tapering and strongly setose on apical quarter. Labella at least 4 times as long as wide, somewhat concave medially, tapered apically, with several strong setae towards apex and densely setose along entire medial surface. Antenna (Fig 5F): number of flagellomeres 8, occasionally 9 in both sexes (n = 49♀, 32♂); number occasionally differs between antennae of same individual; flagellomeres cylindrical, barrel-shaped, about 1.3 times as long as wide in female, 1.7 times as long as wide in male; apical flagellomere slightly tapered.

Thorax: Brownish-orange, covered by black and white scales creating three longitudinal black stripes along dorsum separated by thinner white stripes; pleura with white scales. Anepimeron with a group of 5–18 setae. Empodia about as long as bend in claw. Wing length: 1.06–1.44 mm in males (n = 17), 1.14–1.44 mm in females (n = 21). Halter white.

Female abdomen: General color brownish-orange covered by a mixture of reddish-brown scales speckled with black scales and narrow transverse strip of white scales along posterior margin. Sclerites of tergite 8 (Fig 7F) strongly pigmented, with wide anterior area narrowing gradually towards posterior, with 3–7 setae on widened posterior section. Eighth tergite 1.46–2.16 times as long as seventh tergite (n = 21). Ovipositor 5.05–7.04 times as long as eighth tergite (n = 18). Dorso-lateral plate on cercal segment with 10–14 strong, straight setae.

Larva (third instar). Antennae about twice as long as wide. Spatula (Fig 9G) bidentate, with narrow gap between teeth; shaft pigmentation evanescent posteriorly. On each side of spatula 4

asetose lateral papillae, one of which farther laterally than others. Eighth abdominal segment with 2 setose papillae on each side.

Pupa (Fig 4F). Antennal bases form short bi-dentate horns; lateral lobe slightly longer than median lobe; both tapered, pointed ventroapically. Horns widely separated on vertex by horizontal ridge.

Distribution. Widespread in Europe to Siberia. Introduced into and presently widespread in North America.

Material examined. The type series of *Ozirhincus millefolii* (Wachtl) could not be found in the Natural History Museum in Vienna (NHMW), where it was supposedly deposited (Peter Sehnal, pers. comm.) and is considered lost. We therefore designate a Neotype for it as follows: NEOTYPE: ♀, Germany, NRW, Ägedienberg, 11.viii.2011, N. Dorchin, reared from *Achillea millefolium*. The neotype is mounted on a permanent microscope slide in euparal and deposited in TAUI.

OTHER MATERIAL EXAMINED: (all from *Achillea millefolium*): 2♀, Germany, Remagen, 14.v.1907, collector not specified (NHMW); 2♀, 1♂, collection details not given, from Mik collection (NHMW); 1♀, 1♂, UK, Slough, viii.1938, HF Barnes, BMNH(E) 1633185–6; 1♀, 1♂, Czech Republic, Silesia, Roudno, 30.vii.1958, M. Skuhravá (Skuhravá collection); 2♀, 2♂, Austria, Waldviertel, Pargatstetten, 700m a.s.l., 2.ix.1991, Marcela Skuhravá (in ethanol); 5♀, 3♂ (on slides), 3♀ (pinned), Germany, NRW, Ägedienberg, 11.viii.2011, N. Dorchin (same data as neotype) (1♀ and 1♂ ZMBH, others TAUI); 6♀, 3♂, 4 larvae, Germany, NRW, Wahner Heide, 11.viii.2011, N. Dorchin; 7♀, 11♂ (on slides), 4♀, 2♂ (pinned), Germany, NRW, Leverkusen, 14.viii.2011, E. Diehl (2♀, 2♂ ZFMK, others TAUI); 5♀, 6♂, UK, Ripley, Surrey, Gravel Pits, 01.viii.2011, KM. Harris; 17♀, 4♂,USA, PA, Lewisburg, 15.vii.2012, N. Dorchin.

Biology. The life history of this species is similar to that of *O. longicollis*. Its main host plant is *Achillea millefolium*, from which it can be reared in great numbers in July-August in Europe and the USA.

Remarks. This species was described in detail by Wachtl [31] from Austria, with comparison to *Clinorrhyncha chrysanthemi*, but not to *Ozirhincus longicollis*, which was placed in a different genus at the time. It was probably introduced into North America with the seeds of its main host plant, *Achillea millefolium*, during colonial times [4], and was first recognized there by Felt [32], who described it as *Clinorhyncha filicis*, and again in 1908 as *C. kanerensis* [33]. These two species were later synonymized under *O. millefolii* by Gagné [5].

Ozirhincus millefolii is usually smaller than other *Ozirhincus* spp., the scale covering on its thorax and abdomen is largely reddish-brown rather than strictly black-and-white, and it is easily distinguishable from the remaining three species by having only 8–9 antennal flagellomeres, and by the position of the antennal horns in the pupa, which are widely splayed. Möhn [12] listed *Santolina chamaecyparissus* as a host plant based on larvae he found in dried herbarium material from France but this plant genus was not verified as a host in the present study.

Ozirhincus trichatus Möhn was described from *Achillea fragrantissima* based on three larvae, which were extracted from herbarium material originally collected in Syria, near Damascus [12]. *Achillea fragrantissima* is found along the eastern Mediterranean region, from Syria, through Jordan and Israel to Egypt, as well as in the Arabian Peninsula. It is a common and widespread plant in Israel, and we have sampled it many times at different localities and dates in an effort to rear *Ozirhincus* from it, without success. We therefore suspect that the identification of the host plant might have been erroneous. Because morphological characters of the larvae are generally unreliable for distinguishing among *Ozirhincus* species, it is currently impossible to verify that *O. trichatus* is a valid species rather than a synonym of *O. millefolii*, which has been recorded by Möhn [12] from numerous *Achillea* species. In view of these facts,

we make *O*. *trichatus* a nomen dubium until adults are reared from *A*. *fragrantissima* that can be identified to species with certainty.

Discussion

Species delimitation and host-plant ranges in *Ozirhincus* have been unclear for over a century, mainly due to complex host associations and the fact that the only revision of the genus [11], [12] was based on the mostly uninformative larvae. As noted by Barnes et al. [10], resolving the taxonomic issues in *Ozirhincus* necessitated a thorough comparative study of specimens from a wide array of host plants. In the present work we combined such a study with molecular methods in order to infer the phylogeny of the genus and provide, for the first time, discrete characters for recognizing the species within it. We found that *Ozirhincus* includes four clearly defined species that can be distinguished from each other based on morphological characters of adults and pupae. Our molecular data supported the morphological findings and corroborated our taxonomic conclusions. It is noteworthy that the most useful morphological character we found is the number of antennal flagellomeres, which is generally considered an unreliable character in the supertribe Lasiopteridi (to which *Ozirhincus* belongs) [22]. While in other Lasiopteridi flagellomere numbers may vary among individuals within the same species (e.g., [6], [34–38]), we showed that in *Ozirhincus* flagellomere numbers are consistent within a species and are diagnostic.

The most conspicuous morphological character of *Ozirhincus* is the elongate proboscis, although we do not know what function it serves. While it is assumed that the short-lived adults of most cecidomyiids do not feed [4], some are known to consume nectar or pollen from flowers, and may effect pollination (e.g., [39], [40]). This is in contrast to cecidomyiid species that effect pollination by using flowers as oviposition sites rather than a food source for the adults (e.g., [41–43]). In the case of *Ozirhincus*, because the females spend some time on the inflorescences when ovipositing into flowers, they end up carrying some pollen grains on their body, and possibly contribute towards pollination, but we do not know if they actually feed on the flowers by inserting their proboscis into them. If the adults do feed on nectar or pollen, one might expect that the length of their proboscis will correlate with the length of the flowers of their hosts, but this is not the case. *Ozirhincus anthemidis*, whose main hosts are *Chrysanthemum* species, has the shortest proboscis in the genus, whereas *O*. *millefolii* and *O*. *longicollis* have much longer proboscises, yet they are associated with the shorter flowers of *Achillea* and *Anthemis* species. Determining if and how *Ozirhincus* adults use the flowers of their host plants as a food source requires more study.

Ozirhincus, which evolved for larval development in achenes of Anthemideae host plants, can be regarded as an offshoot of the closely related genus *Lasioptera*. Möhn [12] argued that a short proboscis, as seen in *O*. *anthemidis*, is the ancestral state in the genus, because it represents a transitional state between this genus and *Lasioptera*. The molecular analysis conducted in the present study, while clearly supporting the validity of four species in *Ozirhincus*, was unable to resolve the phylogenetic relationships among them, and ancestral states reconstruction does not offer insight into the evolution of proboscis size. Nevertheless, a position of the short-snouted *O*. *anthemidis* at the base of the tree would be the most parsimonious inference, with the very long snout of *O*. *longicollis* representing a derived state. Morphologically, *Ozirhincus* is much closer to *Lasioptera* than to other Lasiopterini, as has also been confirmed by our molecular data. However, it is not clear if *Ozirhincus* evolved from Asteraceae-feeding species within the biologically diverse *Lasioptera* (currently with 130 species) because most of the relevant *Lasioptera* species are associated with different Asteraceae tribes. Of the six *Lasioptera* species that develop in Anthemideae, five are associated with *Artemisia*, which is not used by

Table 2.

ID on tree	Taxon	Collecting location	Host plant	Collecting date	Morphological voucher ID	DNA voucher ID	GenBank Acc. # COI	GenBank Acc. # 16S
Baldratia sp.	*Baldratia sp.*	Israel: Mizpe Yeriho	*Suaeda asphaltica*	16.ii.2014	178092		KP399947	
Careopalpis sp.	*Careopalpis sp.*	Israel: Enot Zuqim	*Suaeda fruticosa*	2.iii.2014	178909		KP399948	
Stefaniola sp.	*Stefaniola sp.*	Israel: Nahal Zeruya	*Suaeda fruticosa*	16.ii.2014	178094		KP399949	
Lasioptera carophila	*Lasioptera carophila*	Israel: Kefar Hahoresh	*Phoeniculum vulgare*	12.x.2009	133693	ZFMK-DNA-0155668040	KP399946	
Her12-5	*Ozirhincus anthemidis*	Israel: Herzeliya	*Chrysanthemum coronarium*	19.iv.2012	137023–137025	ZFMK-DNA-0100405620	KP399913	KR338912
Her12-6	*Ozirhincus anthemidis*	Israel: Herzeliya	*Chrysanthemum coronarium*	19.iv.2012	137023–137025	ZFMK-DNA-0100405619	KP399916	KR338915
Her12-7	*Ozirhincus anthemidis*	Israel: Herzeliya	*Chrysanthemum coronarium*	19.iv.2012	137023–137025	ZFMK-DNA-0100405618	KP399919	KR338916
KH12-8	*Ozirhincus anthemidis*	Israel: Kefar Hahoresh	*Chrysanthemum coronarium*	28.iv.2012	137026–137027	ZFMK-DNA-0100405617	KP399922	KR338918
KH12-9	*Ozirhincus anthemidis*	Israel: Kefar Hahoresh	*Chrysanthemum coronarium*	28.iv.2012	137026–137027	ZFMK-DNA-0100405616	KP399925	KR338919
KH12-10	*Ozirhincus anthemidis*	Israel: Kefar Hahoresh	*Chrysanthemum coronarium*	28.iv.2012	137026–137027	ZFMK-DNA-0100405615	KP399927	KR338921
NeA12-11	*Ozirhincus anthemidis*	Israel: Newe Ativ	*Anthemis tinctoria*	25.v.2012	153147	ZFMK-DNA-0100405614	KP399929	KR338923
NeA12-12	*Ozirhincus anthemidis*	Israel: Newe Ativ	*Anthemis tinctoria*	25.v.2012	153147	ZFMK-DNA-0100405613	KP399931	KR338924
NeA12-13	*Ozirhincus anthemidis*	Israel: Newe Ativ	*Anthemis tinctoria*	25.v.2012	153147	ZFMK-DNA-0100405612	KP399933	KR338926
Bu12-14	*Ozirhincus anthemidis*	Israel: Mt. Hermon	*Anthemis rascheyana*	25.v.2012	137030–137031	ZFMK-DNA-0100404594	KP399934	KR338927
Bu12-15	*Ozirhincus anthemidis*	Israel: Mt. Hermon	*Anthemis rascheyana*	25.v.2012	137030–137031	ZFMK-DNA-0100405599	KP399935	KR338928
Bu12-16	*Ozirhincus anthemidis*	Israel: Mt. Hermon	*Anthemis rascheyana*	25.v.2012	137030–137031	ZFMK-DNA-0100405600	KP399936	KR338929
Niz13-16	*Ozirhincus anthemidis*	Israel: Nahal Nizzana	*Anthemis retusa*	12.v.2013	151802	ZFMK-DNA-0155668041	KP399937	KR338930
Niz13-17	*Ozirhincus anthemidis*	Israel: Nahal Nizzana	*Anthemis retusa*	12.v.2013	151802	ZFMK-DNA-0155668042	KP399939	KR338932
Ben12-18	*Ozirhincus anthemidis*	Israel: Ma'agar Bental	*Anthemis bornmuelleri*	14.v.2012	137036	ZFMK-DNA-0100405602	KP399940	KR338933
Niz13-18	*Ozirhincus anthemidis*	Israel: Nahal Nizzana	*Anthemis retusa*	12.v.2013	151802	ZFMK-DNA-0155667693	KP399941	KR338934
Ben12-19	*Ozirhincus anthemidis*	Israel: Ma'agar Bental	*Anthemis bornmuelleri*	14.v.2012	137036	ZFMK-DNA-0100405603	KP399942	KR338935
Ben12-20	*Ozirhincus anthemidis*	Israel: Ma'agar Bental	*Anthemis bornmuelleri*	14.v.2012	137036	ZFMK-DNA-0100405604	KP399943	KR338936
Ben12-21	*Ozirhincus anthemidis*	Israel: Ma'agar Bental	*Anthemis bornmuelleri*	14.v.2012	137036	ZFMK-DNA-0100405605	KP399944	KR338937
WH11-3	*Ozirhincus hungaricus*	Germany: Wahner Heide	*Tanacetum vulgare*	11.viii.2011	137016, 137018, 137022	ZFMK-DNA-0100405216	KP399906	KR338907
Lev11-4	*Ozirhincus hungaricus*	Germany: Leverkusen	*Tanacetum vulgare*	14.viii.2011	137020, 137021	ZFMK-DNA-0100405217	KP399909	KR338909
Aeg11-5	*Ozirhincus hungaricus*	Germany: Aegidienberg	*Tanacetum vulgare*	11.viii.2011	137017, 137019	ZFMK-DNA-0100405218	KP399912	KR338911

(*Continued*)

Table 2. (*Continued*)

ID on tree	Taxon	Collecting location	Host plant	Collecting date	Morphological voucher ID	DNA voucher ID	GenBank Acc. # COI	GenBank Acc. # 16S
UKS11-7	*Ozirhincus hungaricus*	UK: Send, Woodhill	*Tripleurospermum inodorum*	20.viii.2011	137043–137044	ZFMK-DNA-0100405220	KP399918	
Lev13-10	*Ozirhincus hungaricus*	Germany: Leverkusen	*Tripleurospermum inodorum*	30.viii.2011	137046, 151804	ZFMK-DNA-0155667731	KP399928	KR338922
Lev13-11	*Ozirhincus hungaricus*	Germany: Leverkusen	*Tripleurospermum inodorum*	30.viii.2011	137046, 151804	ZFMK-DNA-0155668036	KP399930	
UKP13-7	*Ozirhincus hungaricus*	UK: Papercourt Lock	*Tanacetum vulgare*	18.viii.2011	153704	ZFMK-DNA-0155667692	KP399920	
UKP13-8	*Ozirhincus hungaricus*	UK: Papercourt Lock	*Tanacetum vulgare*	18.viii.2011	153704	ZFMK-DNA-0155668033	KP399923	
UK11-6	*Ozirhincus longicollis*	UK: Send, Woodhill	*Tripleurospermum inodorum*	20.viii.2011	137043–137044	ZFMK-DNA-0100404551	KP399915	KR338914
Lev11-8	*Ozirhincus longicollis*	Germany: Leverkusen	*Tripleurospermum inodorum*	30.viii.2011	137046, 151804	ZFMK-DNA-0100405221	KP399921	KR338917
Ben13-1	*Ozirhincus longicollis*	Israel: Ma'agar Bental	*Anthemis bornmuelleri*	25.v.2012	137034	ZFMK-DNA-0155622342	KP399902	KR338904
Ben13-2	*Ozirhincus longicollis*	Israel: Ma'agar Bental	*Anthemis bornmuelleri*	25.v.2012	137034	ZFMK-DNA-0155622343	KP399905	KR338906
Ben13-3	*Ozirhincus longicollis*	Israel: Ma'agar Bental	*Anthemis bornmuelleri*	25.v.2012	137034	ZFMK-DNA-0155622344	KP399908	KR338908
UK13-4	*Ozirhincus longicollis*	UK: Send, Woodhill	*Tripleurospermum inodorum*	25.viii.2011	153248	ZFMK-DNA-0155622345	KP399911	KR338910
UK13-9	*Ozirhincus longicollis*	UK: Send, Woodhill	*Tripleurospermum inodorum*	20.viii.2011	137043, 137044	ZFMK-DNA-0155668034	KP399926	KR338920
UK13-12	*Ozirhincus longicollis*	UK: Newlands Corner	*Leucanthemum vulgare*	15.ix.2011	153149	ZFMK-DNA-0155668037	KP399932	KR338925
Ben12-17	*Ozirhincus longicollis*	Israel: Ma'agar Bental	*Anthemis bornmuelleri*	14.v.2012	137036	ZFMK-DNA-0100405601	KP399938	KR338931
Ben12-22	*Ozirhincus longicollis*	Israel: Ma'agar Bental	*Anthemis bornmuelleri*	14.v.2012	137036	ZFMK-DNA-0100405606	KP399945	KR338938
Lev11-1	*Ozirhincus millefolii*	Germany: Wahner Heide	*Achillea millefolium*	11.viii.2011	137010, 137011, 137013	ZFMK-DNA-0100405227	KP399900	KR338902
US12-1	*Ozirhincus millefolii*	USA: PA, Lewisburg	*Achillea millefolium*	15.vii.2012	133890	ZFMK-DNA-0100405633	KP399901	KR338903
Lev11-2	*Ozirhincus millefolii*	Germany: Leverkusen	*Achillea millefolium*	14.viii.2011	137012	ZFMK-DNA-0100405215	KP399903	KR338905
US12-2	*Ozirhincus millefolii*	USA: PA, Lewisburg	*Achillea millefolium*	15.vii.2012	133890	ZFMK-DNA-0100405634	KP399904	
US12-3	*Ozirhincus millefolii*	USA: PA, Lewisburg	*Achillea millefolium*	15.vii.2012	133890	ZFMK-DNA-0100405622	KP399907	
Aeg12-4	*Ozirhincus millefolii*	Germany: Aegidienberg	*Achillea millefolium*	11.viii.2011	137007	ZFMK-DNA-0100405621	KP399910	
UK13-5	*Ozirhincus millefolii*	UK: Ripley, Gravel Pitts	*Achillea ptarmica*	2.viii.2011	153150	ZFMK-DNA-0155622346	KP399914	KR338913
UK13-6	*Ozirhincus millefolii*	UK: Ripley, Gravel Pitts	*Achillea ptarmica*	2.viii.2011	153150	ZFMK-DNA-0155622347	KP399917	
Lev11-9	*Ozirhincus millefolii*	Germany: Leverkusen	*Achillea millefolium*	15.viii.2011		ZFMK-DNA-0100405222	KP399924	

doi:10.1371/journal.pone.0130981.t002

Ozirhincus [1]. The sixth species, *L. francoisi* Kieffer, was recorded from *Achillea millefolium* but we did not rear it in the present study despite repeated collections of this host plant. Further large-scale sampling of *Lasioptera* species was beyond the scope of the present work.

The fact that *Ozirhincus* is limited to host plants of the tribe Anthemideae is of interest given that other cecidomyiid genera, such as the large genus *Rhopalomyia* Rübsaamen, also appear to prefer this tribe [1]. However, within the Anthemideae, no *Ozirhincus* species has ever been reared from *Artemisia*, a genus that hosts more than 170 *Rhopalomyia* species [44], [1]. One of the main goals achieved in the present study, was to clarify the complex pattern of host use in *Ozirhincus*, which had been a major cause for confusion and uncertainty in the taxonomy of this genus. With the exception of *O. millefolii*, each *Ozirhincus* species is now known to use several host-plant species in more than one genus, and each host-plant genus other than *Achillea* is known to support more than one *Ozirhincus* species. This means that a single sample of some plants can yield two *Ozirhincus* species simultaneously, but in such cases the key and descriptions we provided here make it easy to recognize the species.

This work shows that *Ozirhincus* species are oligophagous, similar to some species of *Lasioptera* (e.g., *L. buhri* Möhn, *L. carophila* Löw), but in stark contrast to the mostly monophagous species in the subtribe Baldratiina (e.g., *Baldratia*, *Careopalpis*, and *Stefaniola*) ([6] and Dorchin, unpubl. data). The extent of oligophagy within *Ozirhincus* varies, with species currently known from between 1–4 genera and 3–12 species of host plants. In those species that exhibit the wider host ranges (*O. longicollis* and *O. anthemidis*), we did not find any evidence for host-race formation, as individuals from different host plants were intermixed in our phylogenetic tree. It is noteworthy that the two species with the smaller number of host species are limited to Europe and the Russian Far East, whereas those with a larger number of host species extend all the way to the southern Mediterranean region. Not surprisingly, this observation suggests that having a greater number of host species contributes toward a wider distribution range.

Appendix A

Samples used for analysis of the COI and 16S mitochondrial genes, with GenBank accession numbers (Table 2). The identification of specimens was done by ND. Morphological vouchers are kept at TAUI, and DNA vouchers are kept at ZFMK.

Acknowledgments

We are grateful to Elise Diehl (Museum Koenig, Bonn) and to Alexandra Brodezki (Dept. of Zoology, Tel Aviv University) for their invaluable help in field collecting and laboratory work, to Claudia Etzbauer, Hannah Janssen and Tina Blume (Museum Koenig, Bonn) for help with the molecular work, to Raymond J. Gagné (USDA, SEL, Washington DC) and Neal L. Evenhuis (Bishop Museum, Hawaii) for nomenclatorial advice, to Ofer Cohen and Yuval Sapir (Tel Aviv University) for help in host-plant identifications, and to the following colleagues for lending, imaging, or donating material from the collections under their care: Hans-Peter Tschorsnig (Staatlisches Museum für Naturkunde, Stuttgart), Joachim Ziegler (Natural History Museum, Berlin), Peter Sehnal (Natural History Museum, Vienna), Duncan Sivell (Natural History Museum, London), Marcela Skuhravá (private collection, Prague), and Eddy Dijkstra (private collection, Wageningen). We are greatly indebted to Janet Rowley, Hedy Jansen, Jerry Clough, and CG Magnusson for their kind permission to use their beautiful photos of live *Ozirhincus* adults in this publication and in scientific presentations that were based on this work. RJ Gagné, Junichi Yukawa, and Chris Borkent provided helpful comments on an earlier version of this manuscript. This study was supported by the Israeli Taxonomy Initiative.

Author Contributions

Conceived and designed the experiments: ND JJA KMH LB. Performed the experiments: ND JJA KMH LB. Analyzed the data: ND JJA. Wrote the paper: ND JJA KMH.

References

1. Gagné RJ, Jaschhof M (2014) Update for a Catalog of the Cecidomyiidae (Diptera) of the World. 3rd edition, digital version 2. Available: http://www.ars.usda.gov/SP2UserFiles/Place/80420580/Gagne_2014_World_Cecidomyiidae_Catalog_3rd_Edition.pdf. Accessed 30 December 2014.
2. Yukawa J, Rohfritsch O (2005) Biology and ecology of gall-inducing Cecidomyiidae (Diptera). In: Raman A, Schaefer CW, Withers TM, editors. Biology, ecology, and evolution of gall-inducing arthropods. Enfield: Science Publishers, Inc. pp. 273–304.
3. Redfern M (2011) Plant Galls. London: Harper Collins Publishers.
4. Gagné RJ (1989) The Plant Feeding Gall Midges of North America. New York: Cornell University Press.
5. Gagné RJ (1969) A tribal and generic revision of the Nearctic Lasiopteridi (Diptera: Cecidomyiidae). Ann Entomol Soc Am 62: 1348–1364. PMID: 5374172
6. Dorchin N, Freidberg A, Mokady O (2004) Phylogeny of the Baldratiina (Diptera: Cecidomyiidae) inferred from multiple data sources, and evolutionary patterns in plant-galler relationships. Mol Phylogenet Evol 30: 503–515. PMID: 15012935
7. Rondani C (1840) Sopra alcuni nuovi generi di insetti ditteri. Memoria Seconda per Servire alla Ditterologia Italiana. Parma. 27 pp.
8. Verrall GH (1889) A study in orthography. Wien Entomol Z 8: 121–123.
9. Rübsaamen H (1916) Cecidomyidenstudien IV. Revision der deutschen Oligotropharien und Lasiopterarien nebst Beschreibung neuer Arten. Sitz Gesell Nat Fr Berlin 1915: 485–567.
10. Barnes HF, Arnold MK, Heath GW (1962) The gall midges (Diptera: Cecidomyiidae) of wild ox-eye daisy (*Chrysanthemum leucanthemum* L.) flowers, with the description of a new species. Trans. Soc. British Entomol 15: 1–20.
11. Möhn E (1966) Cecidomyiidae = (Itonididae) In: Lindner E (editor). Die Fliegen der palaearktischen Region II 2, 6 L, Lieferung 269: 1–48.
12. Möhn E (1968) Cecidomyiidae = (Itonididae) In: Lindner E (editor). Die Fliegen der palaearktischen Region II 2, 6 L, Lieferung 273: 49–96.
13. Skuhravá M (1989) Taxonomic changes and records in Palaearctic Cecidomyiidae (Diptera). Acta Entomol Bohemos 86: 202–233.
14. Folmer O, Black M, Hoeh W, Lutz R, Vrijenhoek R (1994) DNA primers for amplification of mitochondrial cytochrome c oxidase subunit I from diverse metazoan invertebrates. Mol Mar Biol Biotech 3: 294–299.
15. Simon C, Frati F, Beckenbach A, Crespi B, Liu H, Flook P (1994) Evolution, weighting, and phylogenetic utility of mitochondrial gene-sequences and a compilation of conserved polymerase chain-reaction primers. Ann Entomol Soc Am 87: 651–701.
16. Astrin JJ & Stüben PE (2008) Phylogeny in cryptic weevils: molecules, morphology and new genera of western Palaearctic Cryptorhynchinae (Coleoptera: Curculionidae). Invertebr Syst 22: 503–522.
17. Hall TA (1999) BioEdit: a user-friendly biological sequence alignment editor and analysis program for Windows 95/98/NT. Nucl Acids Symp Ser 41: 95–98.
18. Posada D, Crandall KA (1998) MODELTEST: testing the model of DNA substitution. Bioinformatics 14: 817–818. PMID: 9918953
19. Lanave C, Preparata G, Saccone C, Serio G (1984) A new method for calculating evolutionary substitution rates. J Mol Evol 20: 86–93. PMID: 6429346
20. Huelsenbeck JP, Ronquist F (2001) MrBayes: Bayesian inference of phylogenetic trees. Bioinformatics 17: 754–755. PMID: 11524383
21. Maddison WP, Maddison DR. 2011. Mesquite: a modular system for evolutionary analysis. Version 2.75. Available at: http://mesquiteproject.org (accessed 22 Apr 2015).
22. Gagné RJ (1994) The Gall Midges of the Neotropical Region. New York: Cornell University Press.
23. McAlpine JF, Peterson BV, Shewell GE, Teskey HJ, Vockeroth JR, Wood DM, editors (1981) Manual of Nearctic Diptera Vol. 1. Research Branch, Agriculture Canada. Monograph No. 27.

24. Rohfritsch O (1997) Morphological and behavioral adaptations of the gall midge *Lasioptera arundinis* (Schiner) (Diptera, Cecidomyiidae) to collect and transport conidia of its fungal symbiont. Tijdschr Entomol 140**:** 59–66.
25. Rohfritsch O (2008) Plants, gall midges, and fungi: a three component system. Entomol Exp Appl 128**:** 208–216.
26. Loew H (1850) Dipt Beitr IV, Posen. 40pp.
27. Gagné RJ, Solinas M (1996) The Rondani Cecidomyiidae (Diptera). Insect Mundi 10: 69–76.
28. Winnertz J (1853) Beitrag zu einer Monographie der Gallmücken. Linn Entomol 8: 154–322.
29. Kieffer JJ (1898) Synopse des cécidomyies d'Europe et d'Algérie décrites jusqu'à ce jour. Bull Soc Hist Nat Metz (2:) 8: 1–64.
30. Kieffer JJ (1889) Neue Beiträge zur Kenntniss der Gallmücken. Entomol Nach 15: 208–212.
31. Wachtl FA (1884) Eine neue und eine verkannte Cecidomyide. Wien Entomol Z 3: 161–166.
32. Felt EP (1907) New species of Cecidomyiidae. New York State Education Department, New York.
33. Felt EP (1908) Appendix D. pp. 286–422, 489–510, in: 23d report of the State Entomologist on injurious and other insects of the State of New York 1907. N Y State Mus Bull 124: 5–541.
34. Dorchin N, Freidberg A (2008) The Chenopodiaceae gall midges (Diptera: Cecidomyiidae) of the Na'aman salt marsh, Israel. Zootaxa 1937: 1–22.
35. Dorchin N, Freidberg A (2011) The gall midges (Diptera: Cecidomyiidae) of Apiaceae in Israel. Zootaxa 3044: 28–48.
36. Dorchin N, McEvoy MV, Dowling TA, Abrahamson WG, Moore JG (2009). Revision of the goldenrod-feeding Rhopalomyia (Diptera: Cecidomyiidae) in North America. Zootaxa 2152: 1–35.
37. Gagné RJ, Graney L (2014) *Piceacecis* (Diptera: Cecidomyiidae), a new genus for a non-native pest of Norway spruce from Europe and its North American relative. Proc Entomol Soc Wash 116: 378–393.
38. Gagné RJ, Moser JC (2013) The North American gall midges (Diptera: Cecidomyiidae) of hackberries (Cannabaceae: *Celtis* spp.). Mem Am Entomol Soc 49: 1–103.
39. Yuan LC, Luo YB, Thien LB, Fan JH, Xu HL, Yukawa J, et al. (2008). Pollination of *Kadsura longipedunculata* (Schisandraceae), a monoecious basal angiosperm, by female, pollen-eating *Megommata* sp. (Cecidomyiidae: Diptera) in China. Biol J Linn Soc 93: 523–536.
40. Yukawa J, Sato S, Xu HL, Tokuda M (2011) Description of a new species of the genus *Resseliella* (Diptera: Cecidomyiidae), a pollinator of *Kadsura longipedunculata* (Schisandraceae) in China, with comments on its flower-visiting habits. Entomol Sci 14: 297–303.
41. Feil JP (1992) Reproductive ecology of dioecious *Siparuna* (Monimiaceae) in Ecuador–A case of gall midge pollination. Bot J Linn Soc 110: 171–203.
42. Luo SX, Chaw S, Zhang D, Renner SS (2010). Flower heating following anthesis and the evolution of tall midge pollination in Schisandraceae. Am J Bot 97: 1220–1228. doi: 10.3732/ajb.1000077 PMID: 21616873
43. Vislobokov NA, Galinskaya TV, Degtjareva GV, Valiejo-Roman CM, Samigullin TH, Kuznetsov AN, et al. (2014) Pollination of Vietamese *Aspidistra xuansonensis* (Asparagaceae) by female Cecidomyiidi flies: Larvae of pollinator feed on fertile pollen in anthers of anthetic bisexual flowers. Am J Bot 101: 1519–1531. doi: 10.3732/ajb.1400359 PMID: 25253712
44. Jones RG, Gagné RJ, Barr WF (1983) Biology and taxonomy of the *Rhopalomyia* gall midges (Diptera: Cecidomyiidae) of *Artemisia tridentata* Nuttall (Compositae) in Idaho. Contr Am Entomol Inst 21: 1–79.
